# National trends in type 2 diabetes mellitus stratified by central adiposity using waist-to-height ratio in South Korea, 2005–2022

**DOI:** 10.1038/s41598-024-75002-2

**Published:** 2024-10-16

**Authors:** Hyunjee Kim, Seoyoung Park, Jaeyu Park, Yejun Son, Soeun Kim, Yesol Yim, Hyesu Jo, Kyeongmin Lee, Yi Deun Jeong, Jiyeon Oh, Hanseul Cho, Damiano Pizzol, Jiyoung Hwang, Lee Smith, Dong Keon Yon

**Affiliations:** 1 Center for Digital Health, Medical Science Research Institute, Kyung Hee University Medical Center, Kyung Hee University College of Medicine, 23 Kyungheedae–ro, Dongdaemun–gu, Seoul, 02447 South Korea; 2 Department of Precision Medicine, Kyung Hee University College of Medicine, Seoul, South Korea; 3 Department of Regulatory Science, Kyung Hee University, Seoul, South Korea; 4 Department of Medicine, Kyung Hee University College of Medicine, Seoul, South Korea; 5 Health Unit, Eni, Maputo, Mozambique; 6 Health Unit, Eni, San Donato Milanese, Italy; 7 Centre for Health, Performance and Wellbeing, Anglia Ruskin University, East Rd, Cambridge, CB1 1PT UK; 8 Department of Pediatrics, Kyung Hee University College of Medicine, Seoul, South Korea

**Keywords:** Central adiposity, Prevalence, Type 2 diabetes mellitus, South Korea, Endocrinology, Endocrine system and metabolic diseases

## Abstract

Studies investigating the association between type 2 diabetes mellitus and central adiposity are lacking. Therefore, this study aimed to investigate trends in type 2 diabetes mellitus stratified by central adiposity using waist-to-height ratio (WHtR). Trends in type 2 diabetes mellitus were examined by central adiposity, using WHtR, with data from the Korea National Health and Nutrition Examination Survey (2005–2022). Individuals aged 30 years and over who participated in the survey were selected. Type 2 diabetes mellitus was identified based on serum glucose or HbA1c levels, the use of diabetes medications, or a prior diagnosis by a physician. Weighted β-coefficients or odd ratios (ORs) with 95% confidence intervals (CIs) were calculated to assess changes in disease prevalence. A total of 79,368 participants were included in the database (female: 45,163 [56.9%]). from 2005 to 2022, the prevalence of type 2 diabetes mellitus increased from 3.3 to 5.8% in the healthy central adiposity group, from 11.2 to 17.1% in the increased central adiposity group, and from 18.0 to 26.7% in the high central adiposity group. Males, older population, lower education level, lower household income, and smoking are associated with a higher risk of type 2 diabetes. In the high central adiposity group, overweight and obese individuals had higher susceptibility than underweight or normal-weight individuals, with ORs of 5.85 (95% CI, 2.54–13.47) and 8.24 (3.79–17.94), respectively. The prevalence of type 2 diabetes mellitus has increased in all central adiposity groups in the past decade. This underscores the need for tailored interventions to address disparities and improve diabetes management in at-risk populations.

**Supplementary Information:**

The online version contains supplementary material available at 10.1038/s41598-024-75002-2.

## Introduction

Central adiposity, defined as the excessive accumulation of fat in the abdominal region, has exhibited a close association with various metabolic disorders, including type 2 diabetes mellitus^1,2^. High central adiposity is a crucial risk factor for the development of insulin resistance and other metabolic abnormalities that can lead to chronic health conditions; therefore, the increase in its prevalence is alarming^3,4^. The global prevalence of diabetes has increased steadily^5^. A similar trend has been observed in South Korea, with the prevalence of diabetes among adults aged ≥ 30 years increasing from 8.6% in 2001 to 16.7% in 2020^6^. This global burden highlights the urgent need to identify factors contributing to high central adiposity and develop effective strategies for preventing and managing the increasing prevalence of type 2 diabetes mellitus.

The waist-to-height ratio (WHtR), a straightforward and effective measure that can predict cardiovascular diseases accurately (including diabetes), is a key indicator used to assess central adiposity^7^. Some studies suggest that WHtR is a more accurate indicator of health risks than the widely used body mass index (BMI)^8^. The applicability of WHtR across various age groups and sexes enhances its clinical relevance and practicality^9^.

We aimed to analyze the prevalence of type 2 diabetes mellitus in Korea from 2005 to 2022 by categorizing individuals by their central adiposity levels as divided by the WHtR. The central adiposity groups were stratified into the healthy, increased, and high central adiposity groups^10^. The findings of this study will (1) provide detailed insights into the effect of elevated levels of central adiposity on the risk of developing type 2 diabetes mellitus, (2) underscore the importance of managing high central adiposity in the prevention and management of type 2 diabetes mellitus, (3) contribute to the formulation of effective public health strategies, (4) and validate the clinical utility of WHtR in health management guidelines in Korea.

## Methods

### Patient selection and data collection

Data from the Korea National Health and Nutrition Examination Survey (KNHANES), conducted by the Korea Disease Control and Prevention Agency (KDCA) from 2005 to 2022, was examined in this study^11^. Individuals aged 30 years and over who participated in the survey were selected based on the criteria used in previous studies^12,13^. Data regarding sex, age, region of residence, central adiposity group, BMI group, education level, household income, and smoking status were collected for each participant^14^. A nationally representative sample of 79,368 participants was selected, and the prevalence of type 2 diabetes mellitus was evaluated and stratified by the central adiposity group. The survey, conducted over the span of 18 years, included the following participant counts by year group: 6,960 in 2005–2007, 16,812 in 2008–2010, 14,269 in 2011–2013, 13,408 in 2014–2016, 13,408 in 2017–2019, 4,444 in 2020, 4,421 in 2021, and 4,127 in 2022.

The research protocol was approved by the Institutional Review Boards of the KDCA (2007-02CON-04-P, 2008-04EXP-01-C, 2009-01CON-03-2C, 2010-02CON-21-C, 2011-02CON-06-C, 2012-01EXP-01-2C, 2013-07CON-03-4C, 2013-12EXP-03-5C, 2018-01-03-P-A, 2018-01-03-C-A, 2018-01-03-2C-A, 2018-01-03-5C-A, and 2018-01-03-4C-A) and the local law of the Act (Article 2, Paragraph 1), and Enforcement Regulation (Article 2, Paragraph 2, Item 1) of the Bioethics and Safety Act, Government of Korea. Written informed consent was obtained from all participants before commencing the study. The study protocol adhered to the ethical guidelines established by the relevant national and Institutional Review Boards for human research, as well as the 1975 Helsinki Declaration as amended in 2008. Furthermore, the KNHANES provides public access to its data; therefore, the data can be used for diverse epidemiological investigations.

### Definition of type 2 diabetes mellitus

We aimed to examine the annual variations in the prevalence of type 2 diabetes mellitus among individuals aged 30 years and over by three central adiposity groups from 2005 to 2022. Diabetes mellitus was defined as a diagnosis confirmed by a physician within the past year (self-reported), the utilization of oral hypoglycemic agents or the administration of insulin injections (self-reported), a glycated hemoglobin (HbA1c) level of 6.5% or higher (direct measurement), or fasting blood glucose levels of 126 mg/dL or higher (direct measurement). Fasting blood glucose and HbA1c levels were measured through blood tests conducted by KDCA, while information regarding medical diagnoses of diabetes and the use of glucose-lowering medications or insulin injections was collected through self-reported surveys^15,16^.

To identify patients with type 2 diabetes among those who meet the above criteria, an age criterion has been established. According to the Korean Diabetes Association, the prevalence of diabetes in Korea significantly increases in individuals aged 30 years and older^6^. Given that approximately 90–95% of diabetes patients worldwide are diagnosed with type 2 diabetes within this age group, this age criterion has been utilized^17,18^. Individuals who meet the aforementioned diabetes criteria but are under the age of 30 are classified as having type 1 diabetes.

### Covariates

The covariates in this study were selected based on relevance to the development of type 2 diabetes mellitus. Sex, age (30–39, 40–49, 50–59, and ≥ 60 years), region of residence (urban and rural), central adiposity groups (underweight, increased adiposity, and high central adiposity), BMI groups (underweight, normal weight, overweight, and obese), education level (based on the highest level of education attained: elementary school or lower, middle school, high school, and college or higher education), household income (lowest, second, third, and highest quartiles)^19^, and smoking status (current, ex-, and nonsmokers) were the covariates used in this study. Information on sex, age, region of residence, education level, household income, and smoking status was collected through self-reported data obtained from the health and examination surveys, while physical measurements, including height, weight, and waist circumference, were obtained through direct measurement. The participants were categorized as underweight (< 18.5 kg/m^2^), normal weight (18.5–22.9 kg/m^2^), overweight (23.0–24.9 kg/m^2^), and obese (25.0–34.9 kg/m^2^) based on their BMI values according to Asia-Pacific guidelines^20^. WHtR, calculated by dividing the waist circumference by height, was used to categorize the participants into healthy central adiposity (WHtR 0.40–0.49), increased central adiposity (WHtR 0.50–0.59), and high central obesity (WHtR ≥ 0.60) groups according to the guidance from the National Institute for Health and Care Excellence^10^. These classifications applied to individuals with a BMI of under 35.0 kg/m^2^ regardless of sex, ethnicity, or muscle mass. Therefore, participants with a BMI above 35.0 kg/m^2^ were excluded.

### Statistical analyses

This study utilized quantitative data from the Korea National Health and Nutrition Examination Survey (KNHANES), categorizing the data into crude with percentage and group-weighted analyses with 95% confidence intervals (CIs) to identify trends in the prevalence of diabetes mellitus by the levels of central adiposity in South Korea. Additionally, a weighted odds ratio (wOR) was calculated for each period to confirm these trends. A weighted linear regression model was applied to compute the β-coefficients for the comparison of periods before and during the COVID–19 pandemic, thus assessing the pandemic’s impact^21–23^.

A stratified analysis was conducted for the following covariates, which were categorized into three groups based on central adiposity classifications: sex, age, region of residence, BMI groups, education level, household income, and smoking status. This analysis facilitated the identification of vulnerable variables associated with central obesity among patients with diabetes mellitus and allowed for an evaluation of the pandemic’s effects. A weighted logistic regression was employed, and the wORs were used to assess the impact of the pandemic. A two-sided p-value of less than 0.05 was considered statistically significant, and all statistical analyses were performed using SAS software (version 9.4; SAS Institute, Cary, NC, USA).

## Results

A total of 160,591 participants were surveyed and included in the KHNANES database between 2005 and 2022. However, individuals aged < 30 years were excluded from the dataset in this study according to the age criteria used in previous studies. In addition, 65,642 participants were excluded owing to data regarding age groups, household income, and weight values being missing. Furthermore, 15,581 participants who were under 30 years old were also excluded. Therefore, the final sample comprised 79,368 individuals (Figure S1).

Table 1 indicates the baseline characteristics of the participants over the 18-year period: sex (34,205 [43.10%] males and 45,163 [56.90%] females), age (30–39 years, 14,830 [18.69%]; 40–49 years, 16,833 [21.21%]; 50–59 years, 17,064 [21.50%]; and ≥ 60 years, 30,641 [38.61%]) and central adiposity groups (healthy central adiposity group, 33,207 [41.84%]; increased central adiposity group, 39,807 [50.16%]; and high central adiposity group, 6,354 [8.01%]).

**Table 1:** Baseline characteristics of Korean adults based on the data obtained from the KNHANES, 2005–2022 (*n* = 79,368).

Variables	Total	Pre-pandemic					During the pandemic		
		2005–2007	2008–2010	2011–2013	2014–2016	2017–2019	2020	2021	2022
Overall, n	79,368	6,960	16,812	14,269	13,408	14,927	4,444	4,421	4,127
Crude rate, n (%)									
Sex, n (%)									
Male	34,205 (43.1)	2,947 (42.3)	7,221 (43.0)	6,028 (42.2)	5,716 (42.6)	6,538 (43.8)	1,988 (44.7)	1,958 (44.3)	1,809 (43.8)
Female	45,163 (56.9)	4,013 (57.7)	9,591 (57.0)	8,241 (57.8)	7,692 (57.4)	8,389 (56.2)	2,456 (55.3)	2,463 (55.7)	2,318 (56.2)
Age, years, n (%)									
30–39	14,830 (18.7)	1,710 (24.6)	3,727 (22.2)	2,725 (19.1)	2,429 (18.1)	2,416 (16.2)	675 (15.2)	555 (12.6)	593 (14.4)
40–49	16,833 (21.2)	1,794 (25.8)	3,792 (22.6)	2,913 (20.4)	2,710 (20.2)	3,079 (20.6)	884 (19.9)	878 (19.9)	783 (19.0)
50–59	17,064 (21.5)	1,358 (19.5)	3,436 (20.4)	3,166 (22.2)	2,981 (22.3)	3,330 (22.3)	996 (22.4)	938 (21.2)	859 (20.8)
≥60	30,641 (38.6)	2,098 (30.1)	5,857 (34.8)	5,465 (38.3)	5,288 (39.4)	6,102 (40.9)	1,889 (42.5)	2,050 (46.3)	1,892 (45.8)
Region of residence, n (%)									
Urban	61,338 (77.3)	5,099 (73.3)	12,294 (73.1)	11,194 (78.4)	10,699 (79.8)	11,992 (80.3)	3,504 (78.8)	3,371 (76.2)	3,185 (77.2)
Rural	18,030 (22.7)	1,861 (26.7)	4,518 (26.9)	3,075 (21.6)	2,709 (20.2)	2,935 (19.7)	940 (21.2)	1,050 (23.8)	942 (22.8)
Central adiposity^**a**^, n (%)									
Healthy central adiposity	33,207 (41.8)	2,934 (42.2)	7,422 (44.1)	6,433 (45.1)	5,542 (41.3)	6,161 (41.3)	1,495 (33.6)	1,637 (37.0)	1,583 (38.4)
Increased central adiposity	39,807 (50.2)	3,496 (50.2)	8,132 (48.4)	6,878 (48.2)	6,806 (50.8)	7,581 (50.8)	2,482 (55.9)	2,296 (51.9)	2,136 (51.7)
High central adiposity	6,354 (8.0)	530 (7.6)	1,258 (7.5)	958 (6.7)	1,060 (7.9)	1,185 (7.9)	467 (10.5)	488 (11.1)	408 (9.9)
BMI group^b^, n (%)									
Underweight	1,632 (2.1)	145 (2.1)	377 (2.2)	282 (2.0)	269 (2.0)	287 (1.9)	81 (1.8)	97 (2.2)	94 (2.3)
Normal weight	29,811 (37.6)	2,579 (37.1)	6,486 (38.6)	5,486 (38.4)	5,029 (37.5)	5,601 (37.5)	1,545 (34.8)	1,557 (35.2)	1,528 (37.0)
Overweight	19,859 (25.0)	1,834 (26.3)	4,252 (25.3)	3,617 (25.3)	3,352 (25.0)	3,667 (24.6)	1,074 (24.2)	1,083 (24.5)	980 (23.7)
Obese	28,066 (35.3)	2,402 (34.5)	5,697 (33.9)	4,884 (34.3)	4,758 (35.5)	5,372 (36.0)	1,744 (39.2)	1,684 (38.1)	1,525 (37.0)
Level of education, n (%)									
Elementary school or lower education	16,710 (21.1)	4,246 (61.0)	5,032 (29.9)	3,724 (26.1)	3,054 (22.8)	2,953 (19.8)	728 (16.4)	834 (18.9)	713 (17.3)
Middle school	19,855 (25.0)	1,271 (18.2)	2,248 (13.4)	1,878 (13.1)	1,737 (13.0)	1,779 (11.9)	539 (12.1)	514 (11.6)	422 (10.2)
High school	21,067 (26.5)	833 (12.0)	5,054 (30.1)	4,304 (30.2)	3,868 (28.8)	4,339 (29.1)	1,337 (30.1)	1,301 (29.4)	1,210 (29.3)
College or higher education	21,736 (27.4)	610 (8.8)	4,478 (26.6)	4,363 (30.6)	4,749 (35.4)	5,856 (39.2)	1,840 (41.4)	1,772 (40.1)	1,782 (43.2)
Household income, n (%)									
Lowest quartile	21,284 (26.8)	1,676 (24.1)	3,767 (22.4)	3,031 (21.2)	2,710 (20.2)	3,012 (20.2)	807 (18.2)	884 (20.0)	823 (19.9)
Second quartile	10,388 (13.1)	1,723 (24.8)	4,187 (24.9)	3,712 (26.0)	3,364 (25.1)	3,712 (24.9)	1,076 (24.2)	1,071 (24.2)	1,010 (24.5)
Third quartile	22,246 (28.0)	1,763 (25.3)	4,453 (26.5)	3,706 (26.0)	3,643 (27.2)	3,954 (26.4)	1,240 (27.9)	1,208 (27.3)	1,100 (26.7)
Highest quartile	25,450 (32.1)	1,798 (25.8)	4,405 (26.2)	3,820 (26.8)	3,691 (27.5)	4,249 (28.5)	1,321 (29.7)	1,258 (28.5)	1,194 (28.9)
Smoking status, n (%)									
Smoker	17,645 (22.2)	2,020 (29.0)	5,936 (35.3)	2,762 (19.4)	2,382 (17.8)	2,529 (16.9)	716 (16.1)	676 (15.3)	624 (15.1)
Ex-smoker	14,804 (18.7)	848 (12.2)	1,104 (6.6)	3,041 (21.3)	2,954 (22.0)	3,565 (23.9)	1,106 (24.9)	1,120 (25.3)	1,066 (25.8)
Non-smoker	46,919 (59.1)	4,092 (58.8)	9,772 (58.1)	8,466 (59.3)	8,072 (60.2)	8,833 (59.2)	2,622 (59.0)	2,625 (59.4)	2,437 (59.1)
Weighted rate (95% CI)									
Sex, weighted % (95% CI)									
Male	49.04 (48.72 to 49.36)	46.51 (45.48 to 47.54)	49.24 (48.57 to 49.91)	48.56 (47.79 to 49.33)	48.61 (47.83 to 49.39)	49.43 (48.69 to 50.18)	50.01 (48.86 to 51.16)	50.21 (48.71 to 51.71)	50.27 (48.88 to 51.66)
Female	50.96 (50.64 to 51.28)	53.49 (52.46 to 54.52)	50.76 (50.09 to 51.43)	51.44 (50.67 to 52.21)	51.39 (50.61 to 52.17)	50.57 (49.82 to 51.31)	49.99 (48.84 to 51.14)	49.79 (48.29 to 51.29)	49.73 (48.34 to 51.12)
Age, years, weighted % (95% CI)									
30–39	22.84 (22.27 to 23.40)	28.49 (26.23 to 30.75)	26.35 (25.01 to 27.68)	24.04 (22.75 to 25.33)	22.52 (21.15 to 23.90)	20.57 (19.29 to 21.85)	19.93 (17.55 to 22.32)	18.69 (16.58 to 20.80)	18.81 (16.43 to 21.19)
40–49	25.67 (25.17 to 26.16)	29.03 (27.18 to 30.89)	27.99 (26.85 to 29.13)	26.74 (25.48 to 27.99)	25.27 (24.21 to 26.33)	24.23 (23.08 to 25.37)	24.01 (21.94 to 26.07)	23.39 (21.33 to 25.46)	22.27 (19.98 to 24.56)
50–59	23.50 (23.05 to 23.96)	19.21 (17.94 to 20.49)	21.71 (20.76 to 22.67)	23.46 (22.40 to 24.52)	24.69 (23.56 to 25.82)	24.59 (23.56 to 25.61)	25.30 (23.16 to 27.44)	23.86 (21.77 to 25.96)	24.42 (22.44 to 26.40)
≥60	27.99 (27.30 to 28.68)	23.27 (21.36 to 25.17)	23.95 (22.63 to 25.27)	25.76 (24.26 to 27.25)	27.52 (25.98 to 29.06)	30.62 (28.84 to 32.40)	30.76 (27.58 to 33.93)	34.06 (30.93 to 37.17)	34.50 (30.91 to 38.08)
Region of residence, weighted % (95% CI)									
Urban	81.21 (79.91 to 82.51)	76.69 (72.83 to 80.55)	78.13 (74.88 to 81.38)	79.22 (75.81 to 82.62)	82.76 (79.84 to 85.68)	83.82 (80.81 to 86.83)	83.98 (78.64 to 89.32)	82.64 (77.34 to 87.95)	83.55 (78.09 to 89.01)
Rural	18.79 (17.49 to 20.09)	23.31 (19.46 to 27.17)	21.87 (18.62 to 25.12)	20.78 (17.38 to 24.19)	17.24 (14.32 to 20.16)	16.18 (13.17 to 19.19)	16.02 (10.68 to 21.36)	17.36 (12.05 to 22.66)	16.45 (10.99 to 21.91)
Central obesity^a^, weighted % (95% CI)									
Healthy central adiposity	44.34 (43.82 to 44.86)	43.97 (42.23 to 45.70)	46.91 (45.78 to 48.05)	47.83 (46.56 to 49.11)	44.93 (43.65 to 46.20)	43.94 (42.77 to 45.12)	36.65 (34.70 to 38.61)	39.80 (37.73 to 41.86)	39.81 (37.68 to 41.95)
Increased central adiposity	48.86 (48.40 to 49.32)	49.22 (47.62 to 50.81)	46.95 (45.94 to 47.95)	46.57 (45.43 to 47.70)	48.53 (47.38 to 49.68)	49.34 (48.31 to 50.38)	54.84 (53.17 to 56.51)	51.47 (49.69 to 53.25)	51.12 (49.26 to 52.98)
High central adiposity	6.79 (6.56 to 7.02)	6.82 (6.02 to 7.62)	6.14 (5.65 to 6.63)	5.60 (5.13 to 6.08)	6.54 (5.99 to 7.10)	6.71 (6.18 to 7.24)	8.51 (7.47 to 9.54)	8.73 (7.60 to 9.87)	9.07 (8.04 to 10.09)
BMI group^b^, weighted % (95% CI)									
Underweight	1.88 (1.77 to 1.99)	1.88 (1.54 to 2.23)	1.95 (1.71 to 2.18)	1.75 (1.48 to 2.01)	1.94 (1.68 to 2.20)	1.80 (1.56 to 2.05)	1.86 (1.28 to 2.44)	1.72 (1.30 to 2.14)	2.24 (1.75 to 2.73)
Normal weight	36.78 (36.36 to 37.20)	36.48 (35.23 to 37.73)	37.77 (36.88 to 38.66)	37.46 (36.41 to 38.50)	37.19 (36.16 to 38.22)	37.01 (36.05 to 37.96)	33.40 (31.66 to 35.14)	35.60 (33.79 to 37.41)	35.18 (33.39 to 36.96)
Overweight	24.97 (24.60 to 25.34)	26.23 (25.16 to 27.31)	25.62 (24.82 to 26.42)	25.35 (24.46 to 26.23)	25.02 (24.15 to 25.89)	24.60 (23.76 to 25.44)	24.79 (23.25 to 26.34)	23.16 (21.60 to 24.73)	23.90 (22.32 to 25.47)
Obese	36.37 (35.94 to 36.80)	35.40 (34.00 to 36.81)	34.67 (33.77 to 35.56)	35.45 (34.45 to 36.45)	35.85 (34.83 to 36.88)	36.59 (35.65 to 37.53)	39.95 (38.13 to 41.77)	39.52 (37.68 to 41.36)	38.69 (36.61 to 40.77)
Level of education, weighted % (95% CI)									
Elementary school or lower education	19.59 (19.13 to 20.05)	53.00 (50.74 to 55.26)	23.05 (21.94 to 24.17)	20.08 (18.97 to 21.18)	17.03 (16.02 to 18.04)	14.64 (13.64 to 15.64)	11.24 (9.67 to 12.82)	12.31 (10.66 to 13.96)	11.27 (9.85 to 12.70)
Middle school	11.61 (11.29 to 11.92)	18.49 (16.89 to 20.09)	12.99 (12.31 to 13.67)	12.49 (11.75 to 13.23)	11.20 (10.51 to 11.89)	10.18 (9.49 to 10.87)	9.51 (8.32 to 10.70)	9.12 (7.94 to 10.29)	8.24 (7.04 to 9.45)
High school	30.70 (30.20 to 31.19)	16.08 (14.58 to 17.59)	33.16 (32.05 to 34.27)	33.77 (32.54 to 35.00)	31.07 (29.92 to 32.23)	30.26 (29.12 to 31.40)	31.51 (29.32 to 33.71)	31.29 (29.37 to 33.20)	29.87 (27.87 to 31.88)
College or higher education	38.10 (37.39 to 38.82)	12.42 (10.70 to 14.15)	30.80 (29.31 to 32.28)	33.67 (32.16 to 35.17)	40.70 (39.04 to 42.36)	44.92 (43.15 to 46.68)	47.74 (44.37 to 51.10)	47.28 (44.35 to 50.22)	50.61 (47.74 to 53.48)
Household income, weighted % (95% CI)									
Lowest quartile	16.50 (16.03 to 16.98)	20.04 (18.22 to 21.86)	17.86 (16.75 to 18.98)	17.29 (16.11 to 18.47)	15.93 (14.84 to 17.03)	16.01 (14.87 to 17.14)	14.02 (11.99 to 16.04)	13.92 (12.16 to 15.68)	15.06 (13.32 to 16.79)
Second quartile	24.67 (24.14 to 25.20)	25.18 (23.36 to 26.99)	25.42 (24.21 to 26.64)	26.93 (25.64 to 28.23)	24.01 (22.78 to 25.25)	24.38 (23.20 to 25.56)	22.38 (20.24 to 24.51)	23.15 (21.02 to 25.28)	22.42 (20.36 to 24.47)
Third quartile	28.78 (28.24 to 29.31)	27.02 (25.39 to 28.66)	28.38 (27.23 to 29.54)	27.84 (26.62 to 29.06)	29.60 (28.19 to 31.01)	28.61 (27.50 to 29.73)	29.73 (27.45 to 32.02)	30.62 (28.49 to 32.74)	29.64 (27.23 to 32.05)
Highest quartile	30.05 (29.32 to 30.78)	27.76 (25.44 to 30.08)	28.33 (26.73 to 29.94)	27.94 (26.39 to 29.50)	30.46 (28.57 to 32.35)	31.00 (29.36 to 32.64)	33.88 (30.62 to 37.14)	32.31 (28.84 to 35.78)	32.89 (30.03 to 35.75)
Smoking status, weighted % (95% CI)									
Smoker	25.32 (24.91 to 25.74)	33.65 (32.25 to 35.05)	40.52 (39.63 to 41.41)	24.55 (23.56 to 25.54)	21.90 (20.95 to 22.85)	19.92 (19.04 to 20.80)	18.70 (17.10 to 20.29)	17.43 (15.87 to 19.00)	17.22 (15.56 to 18.87)
Ex-smoker	20.11 (19.76 to 20.45)	10.97 (10.16 to 11.78)	6.94 (6.39 to 7.49)	21.47 (20.68 to 22.27)	22.55 (21.74 to 23.37)	24.74 (23.95 to 25.53)	26.13 (24.81 to 27.44)	27.29 (25.69 to 28.89)	28.19 (26.51 to 29.86)
Non-smoker	54.57 (54.18 to 54.96)	55.38 (54.15 to 56.61)	52.54 (51.74 to 53.33)	53.98 (53.07 to 54.88)	55.55 (54.64 to 56.46)	55.34 (54.45 to 56.23)	55.18 (53.58 to 56.78)	55.27 (53.27 to 57.28)	54.60 (52.75 to 56.45)

Abbreviations: BMI, body mass index; CI, confidence interval; KNHANES, Korea National Health and Nutrition Examination Survey.

^a^ According to the guidelines from the National Institute for Health and Care Excellence, central adiposity is divided into three groups: healthy central adiposity (waist-to-height ratio 0.40–0.49), increased central adiposity (waist-to-height ratio 0.50–0.59), and high central adiposity (waist-to-height ratio ≥ 0.60).

^b^ According to the Asian-Pacific guidelines, BMI is divided into four groups: underweight (< 18.5 kg/m^2^), normal (18.5–22.9 kg/m^2^), overweight (23.0–24.9 kg/m^2^), and obese (25.0–34.9 kg/m^2^).

**Fig. 1:**
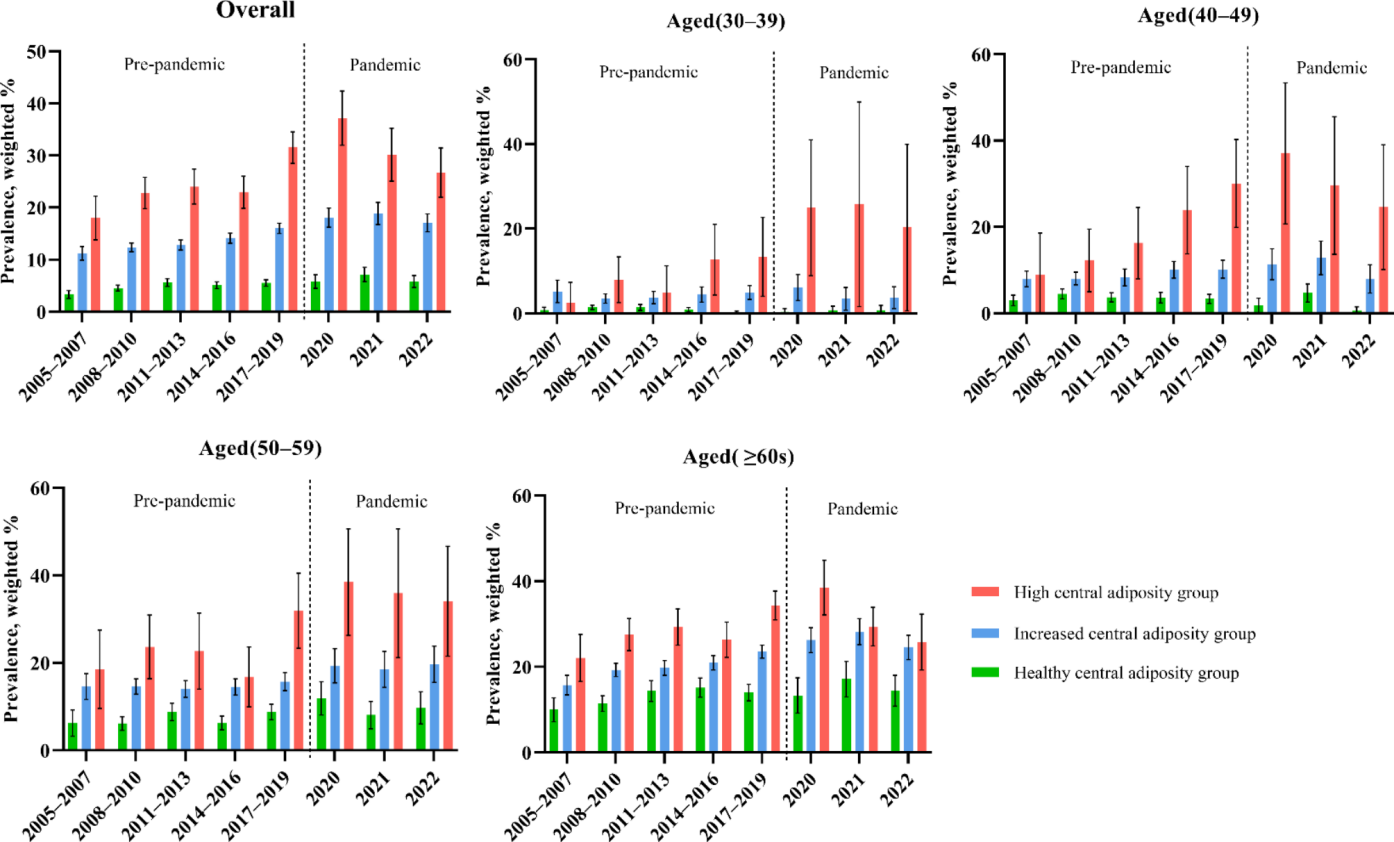
Nationwide trends in the prevalence of type 2 diabetes mellitus among Korean adults stratified by central adiposity groups (healthy, increased, and high central adiposity group), 2005–2022.

Table 2; Fig. 1 present the prevalence of type 2 diabetes mellitus in the central adiposity groups, comparing data for the periods before and during the COVID-19 pandemic from 2005 to 2022. The prevalence of type 2 diabetes mellitus in the healthy, increased, and high central adiposity group increased from 3.32% (95% CI, 2.57 to 4.07) in 2005–2007 to 5.80% (95% CI, 4.67 to 6.93) in 2022, 11.22% (95% CI, 9.91 to 12.53) to 17.07% (95% CI, 15.36 to 18.78), and 18.00% (95% CI, 13.82 to 22.19) to 26.70% (95% CI, 21.96 to 31.44), respectively. Therefore, an increased prevalence of type 2 diabetes mellitus was observed during the study period (Table S1). A specific sub-analysis, comparing the periods of 2017–2019 and 2020–2022 separately, also observed similar trends (Table S2).

**Table 2:** National trends in the prevalence of type 2 diabetes mellitus, stratified by central adiposity groups and β-coefficients of odds ratios before and during the COVID–19 pandemic (weighted % [95% CI]).

Variables		Total	Pre-pandemic					During the pandemic			Trends before the pandemic, β (95% CI)	Trends in the pandemic, β (95% CI)	β_diff_ between 2005–2019 and 2019–2022 (95% CI)
			2005–2007	2008–2010	2011–2013	2014–2016	2017–2019	2020	2021	2022			
Overall	Healthy central adiposity	5.54 (5.20 to 5.88)	3.32 (2.57 to 4.07)	4.54 (3.99 to 5.10)	5.60 (4.85 to 6.34)	5.09 (4.45 to 5.73)	5.53 (4.91 to 6.15)	5.77 (4.46 to 7.09)	7.16 (5.78 to 8.54)	5.80 (4.67 to 6.93)	**2.13 (0.16 to 0.59)**	0.22 (-0.20 to 0.64)	**-1.91 (-2.39 to -1.44)**
	Increased central adiposity	15.66 (15.11 to 16.21)	11.22 (9.91 to 12.53)	12.36 (11.53 to 13.19)	12.81 (11.86 to 13.76)	14.11 (13.14 to 15.08)	16.01 (15.04 to 16.98)	18.05 (16.22 to 19.89)	18.85 (16.72 to 20.98)	17.07 (15.36 to 18.78)	**4.40 (0.85 to 1.53)**	0.38 (-0.28 to 1.04)	**-4.02 (-4.77 to -3.28)**
	High central adiposity	28.18 (26.52 to 29.85)	18.00 (13.82 to 22.19)	22.79 (19.75 to 25.83)	24.04 (20.70 to 27.39)	22.93 (19.86 to 26.00)	31.51 (28.48 to 34.54)	37.16 (31.97 to 42.34)	30.13 (25.05 to 35.21)	26.70 (21.96 to 31.44)	**8.09 (1.62 to 3.80)**	**-2.41 (-4.32 to -0.50)**	**-10.50 (-12.70 to -8.30)**
*Healthy central adiposity* ^a^													
Sex	Male	7.86 (7.26 to 8.45)	5.17 (3.78 to 6.56)	6.17 (5.20 to 7.14)	8.30 (7.04 to 9.56)	7.29 (6.19 to 8.40)	8.28 (7.15 to 9.41)	8.29 (5.92 to 10.65)	9.52 (6.92 to 12.12)	8.31 (6.22 to 10.40)	**3.04 (0.25 to 1.03)**	0.14 (-0.63 to 0.91)	**-2.90 (-3.75 to -2.04)**
	Female	3.56 (3.18 to 3.94)	1.73 (1.08 to 2.38)	2.88 (2.31 to 3.46)	3.01 (2.37 to 3.66)	3.08 (2.43 to 3.74)	3.20 (2.55 to 3.86)	4.03 (2.47 to 5.59)	5.22 (3.77 to 6.67)	3.97 (2.64 to 5.29)	1.61 (-0.01 to 0.44)	0.34 (-0.14 to 0.83)	**-1.26 (-1.80 to -0.73)**
Age, years	30–39	0.84 (0.58 to 1.10)	0.75 (0.10 to 1.41)	1.40 (0.88 to 1.92)	1.39 (0.70 to 2.07)	0.86 (0.32 to 1.40)	0.25 (0.00 to 0.53)	0.38 (0.00 to 1.13)	0.69 (0.00 to 1.66)	0.64 (0.00 to 1.90)	-3.22 (-0.40 to -0.10)	0.15 (-0.25 to 0.54)	**3.36 (2.94 to 3.79)**
	40–49	3.23 (2.74 to 3.71)	2.96 (1.74 to 4.18)	4.48 (3.32 to 5.63)	3.69 (2.65 to 4.73)	3.62 (2.40 to 4.83)	3.33 (2.27 to 4.39)	1.81 (0.16 to 3.45)	4.67 (2.56 to 6.79)	0.57 (0.00 to 1.49)	-1.16 (-0.57 to 0.22)	-0.53 (-1.03 to -0.03)	0.63 (-0.01 to 1.27)
	50–59	8.50 (7.51 to 9.50)	6.22 (3.21 to 9.23)	6.14 (4.60 to 7.68)	8.78 (6.79 to 10.77)	6.25 (4.65 to 7.85)	8.75 (6.98 to 10.52)	11.88 (8.06 to 15.69)	8.06 (4.92 to 11.20)	9.70 (6.05 to 13.35)	2.34 (-0.15 to 1.17)	-0.07 (-1.35 to 1.21)	**-2.41 (-3.85 to -0.97)**
	≥60	14.31 (13.12 to 15.50)	10.01 (7.24 to 12.77)	11.40 (9.57 to 13.24)	14.35 (11.90 to 16.79)	15.13 (12.91 to 17.35)	14.00 (12.07 to 15.94)	13.32 (9.22 to 17.43)	17.12 (12.98 to 21.26)	14.43 (10.83 to 18.03)	**3.34 (0.19 to 1.65)**	0.44 (-0.91 to 1.79)	**-2.90 (-4.43 to -1.36)**
Region of residence	Urban	5.22 (4.85 to 5.59)	3.16 (2.32 to 4.00)	4.30 (3.70 to 4.90)	5.11 (4.33 to 5.88)	5.12 (4.41 to 5.83)	5.15 (4.51 to 5.79)	5.68 (4.17 to 7.18)	6.75 (5.25 to 8.25)	5.03 (3.89 to 6.18)	**2.10 (0.13 to 0.59)**	0.08 (-0.35 to 0.51)	**-2.02 (-2.52 to -1.53)**
	Rural	7.30 (6.39 to 8.21)	3.90 (2.28 to 5.52)	5.63 (4.21 to 7.05)	7.95 (5.81 to 10.08)	4.95 (3.44 to 6.47)	8.02 (5.94 to 10.10)	6.38 (3.80 to 8.97)	9.57 (6.51 to 12.63)	10.71 (6.24 to 15.17)	**3.26 (0.03 to 1.23)**	1.10 (-0.40 to 2.59)	**-2.16 (-3.77 to -0.55)**
BMI group^b^	Underweight or normal weight	5.92 (5.51 to 6.34)	3.50 (2.64 to 4.36)	4.45 (3.82 to 5.09)	5.62 (4.69 to 6.55)	5.23 (4.48 to 5.98)	6.03 (5.28 to 6.79)	5.82 (4.32 to 7.31)	8.14 (6.47 to 9.81)	6.76 (5.38 to 8.14)	**2.91 (0.25 to 0.77)**	0.44 (-0.07 to 0.95)	**-2.46 (-3.04 to -1.89)**
	Overweight	4.39 (3.79 to 4.99)	2.76 (1.25 to 4.28)	4.89 (3.67 to 6.12)	6.02 (4.57 to 7.47)	4.12 (2.89 to 5.34)	4.17 (2.93 to 5.41)	5.61 (2.57 to 8.65)	3.95 (1.87 to 6.02)	2.11 (0.69 to 3.53)	-0.55 (-0.54 to 0.35)	-0.73 (-1.37 to -0.08)	-0.17 (-0.96 to 0.61)
	Obese	4.82 (3.28 to 6.36)	3.36 (0.28 to 6.45)	4.26 (1.58 to 6.94)	3.86 (1.79 to 5.94)	7.40 (3.01 to 11.78)	4.22 (1.84 to 6.60)	5.80 (0.00 to 12.63)	4.37 (0.00 to 9.20)	5.16 (0.00 to 13.18)	2.25 (-0.58 to 1.36)	0.18 (-2.23 to 2.59)	-2.07 (-4.67 to 0.53)
Level of education	Middle school or lower education	10.39 (9.42 to 11.35)	3.30 (2.58 to 4.02)	8.67 (7.27 to 10.07)	10.71 (8.61 to 12.81)	9.92 (7.95 to 11.89)	13.34 (11.04 to 15.65)	12.39 (6.44 to 18.34)	15.91 (11.08 to 20.74)	16.98 (11.17 to 22.79)	**10.27 (1.64 to 2.68)**	1.35 (-0.52 to 3.23)	**-8.92 (-10.86 to -6.97)**
	College or higher education	4.53 (4.17 to 4.89)	3.37 (1.73 to 5.02)	3.33 (2.73 to 3.93)	4.29 (3.58 to 5.00)	4.15 (3.50 to 4.80)	4.32 (3.70 to 4.93)	5.00 (3.70 to 6.29)	6.07 (4.61 to 7.53)	4.61 (3.53 to 5.69)	**1.64 (0.02 to 0.53)**	0.20 (-0.21 to 0.61)	**-1.44 (-1.92 to -0.95)**
Household income	Lowest and second quartile	7.28 (6.67 to 7.88)	4.07 (2.90 to 5.25)	6.40 (5.39 to 7.40)	7.23 (5.98 to 8.48)	7.66 (6.37 to 8.94)	7.97 (6.70 to 9.23)	5.12 (3.04 to 7.19)	10.42 (7.64 to 13.20)	7.41 (5.20 to 9.63)	**3.57 (0.31 to 1.14)**	0.28 (-0.54 to 1.10)	**-3.29 (-4.21 to -2.37)**
	Third and highest quartile	4.68 (4.26 to 5.10)	2.84 (1.88 to 3.79)	3.46 (2.88 to 4.05)	4.55 (3.71 to 5.40)	3.89 (3.19 to 4.58)	4.32 (3.64 to 5.00)	6.04 (4.32 to 7.75)	5.87 (4.19 to 7.56)	5.11 (3.77 to 6.45)	**1.68 (0.02 to 0.50)**	0.23 (-0.27 to 0.73)	**-1.45 (-2.00 to -0.89)**
Smoking status	Smoker or ex-smoker	7.53 (6.93 to 8.13)	4.99 (3.68 to 6.31)	5.81 (4.81 to 6.80)	7.87 (6.57 to 9.16)	7.41 (6.24 to 8.57)	7.78 (6.61 to 8.94)	7.29 (5.09 to 9.49)	9.71 (7.05 to 12.37)	7.96 (5.82 to 10.10)	**3.11 (0.24 to 1.03)**	0.29 (-0.50 to 1.08)	**-2.82 (-3.70 to -1.94)**
	Non-smoker	4.02 (3.62 to 4.41)	2.00 (1.22 to 2.78)	3.37 (2.72 to 4.01)	3.67 (2.95 to 4.38)	3.32 (2.65 to 3.99)	3.85 (3.14 to 4.56)	4.76 (3.05 to 6.47)	5.40 (3.98 to 6.81)	4.31 (2.93 to 5.69)	**1.71 (0.01 to 0.49)**	0.21 (-0.30 to 0.71)	**-1.50 (-2.06 to -0.94)**
*Increased central adiposity* ^a^													
Sex	Male	17.24 (16.41 to 18.06)	13.67 (11.75 to 15.59)	13.62 (12.31 to 14.93)	14.22 (12.82 to 15.63)	15.49 (14.04 to 16.94)	17.10 (15.65 to 18.55)	19.37 (16.57 to 22.17)	21.54 (18.59 to 24.48)	17.82 (15.37 to 20.27)	**3.66 (0.51 to 1.54)**	0.38 (-0.59 to 1.35)	**-3.28 (-4.38 to -2.18)**
	Female	13.74 (13.05 to 14.43)	8.69 (7.29 to 10.09)	11.01 (9.89 to 12.13)	11.30 (10.07 to 12.52)	12.58 (11.37 to 13.79)	14.65 (13.45 to 15.86)	16.26 (13.79 to 18.74)	15.31 (12.75 to 17.88)	16.01 (13.59 to 18.44)	**5.06 (0.88 to 1.72)**	0.31 (-0.58 to 1.20)	**-4.75 (-5.74 to -3.77)**
Age, years	30–39	4.33 (3.51 to 5.15)	5.16 (2.50 to 7.82)	3.49 (2.36 to 4.63)	3.71 (2.26 to 5.17)	4.42 (2.64 to 6.20)	4.93 (3.28 to 6.57)	6.11 (3.05 to 9.16)	3.42 (0.71 to 6.12)	3.70 (1.11 to 6.29)	1.45 (-0.35 to 0.81)	-0.67 (-1.70 to 0.35)	**-2.12 (-3.30 to -0.95)**
	40–49	9.77 (8.75 to 10.79)	7.93 (6.12 to 9.75)	8.04 (6.55 to 9.52)	8.27 (6.31 to 10.23)	10.06 (8.13 to 11.99)	10.20 (8.14 to 12.26)	11.35 (7.78 to 14.93)	12.83 (8.92 to 16.74)	7.94 (4.65 to 11.22)	**3.25 (0.08 to 1.36)**	-0.53 (-1.83 to 0.77)	**-3.78 (-5.23 to -2.33)**
	50–59	16.70 (15.54 to 17.86)	14.57 (11.62 to 17.53)	14.56 (12.80 to 16.31)	14.03 (12.11 to 15.95)	14.46 (12.62 to 16.31)	15.69 (13.62 to 17.76)	19.32 (15.41 to 23.23)	18.50 (14.38 to 22.61)	19.67 (15.50 to 23.83)	1.12 (-0.41 to 1.04)	1.09 (-0.42 to 2.60)	-0.02 (-1.70 to 1.65)
	≥60	23.49 (22.58 to 24.40)	15.74 (13.46 to 18.02)	19.29 (17.74 to 20.84)	19.76 (18.03 to 21.49)	20.94 (19.28 to 22.60)	23.53 (22.00 to 25.05)	26.21 (23.30 to 29.12)	28.19 (25.17 to 31.20)	24.53 (21.69 to 27.37)	**4.96 (1.02 to 2.17)**	0.46 (-0.61 to 1.53)	**-4.50 (-5.71 to -3.29)**
Region of residence	Urban	15.40 (14.76 to 16.04)	11.69 (10.06 to 13.31)	12.68 (11.72 to 13.64)	12.55 (11.48 to 13.62)	13.71 (12.65 to 14.78)	15.32 (14.25 to 16.38)	17.84 (15.76 to 19.92)	17.78 (15.36 to 20.20)	16.99 (14.99 to 19.00)	**3.30 (0.50 to 1.27)**	0.47 (-0.29 to 1.23)	**-2.82 (-3.68 to -1.97)**
	Rural	16.76 (15.63 to 17.89)	9.77 (7.74 to 11.81)	11.33 (9.77 to 12.88)	13.67 (11.56 to 15.78)	15.89 (13.38 to 18.40)	19.13 (16.65 to 21.61)	19.15 (15.48 to 22.81)	23.61 (19.18 to 28.05)	17.42 (14.22 to 20.63)	**8.80 (1.68 to 3.17)**	-0.06 (-1.46 to 1.34)	**-8.86 (-10.45 to -7.28)**
BMI group^b^	Underweight or normal weight	19.10 (17.63 to 20.58)	9.68 (6.68 to 12.68)	14.23 (11.96 to 16.50)	17.05 (14.29 to 19.81)	15.53 (13.08 to 17.98)	18.37 (15.88 to 20.86)	23.76 (18.88 to 28.63)	20.99 (15.75 to 26.23)	24.26 (19.20 to 29.32)	**5.31 (0.65 to 2.38)**	1.46 (-0.39 to 3.32)	**-3.85 (-5.89 to -1.80)**
	Overweight	15.33 (14.39 to 16.26)	12.89 (10.66 to 15.11)	11.43 (9.94 to 12.91)	12.19 (10.63 to 13.74)	14.18 (12.58 to 15.79)	16.09 (14.38 to 17.80)	18.24 (14.58 to 21.90)	17.24 (14.06 to 20.42)	16.70 (13.79 to 19.61)	**4.60 (0.64 to 1.81)**	0.05 (-1.08 to 1.19)	**-4.55 (-5.83 to -3.27)**
	Obese	15.01 (14.27 to 15.75)	10.63 (8.80 to 12.45)	12.39 (11.26 to 13.51)	12.16 (10.91 to 13.40)	13.73 (12.42 to 15.04)	15.39 (14.14 to 16.64)	16.56 (14.11 to 19.01)	19.19 (16.34 to 22.04)	15.51 (13.18 to 17.84)	**4.08 (0.64 to 1.54)**	0.29 (-0.59 to 1.18)	**-3.78 (-4.78 to -2.79)**
Level of education	Middle school or lower education	20.21 (19.38 to 21.04)	12.32 (10.85 to 13.80)	15.35 (14.05 to 16.64)	15.90 (14.33 to 17.48)	18.73 (17.02 to 20.43)	22.61 (20.91 to 24.30)	27.18 (23.55 to 30.81)	27.29 (23.60 to 30.98)	24.44 (20.99 to 27.89)	**8.41 (1.88 to 2.89)**	0.68 (-0.55 to 1.91)	**-7.72 (-9.05 to -6.39)**
	College or higher education	13.46 (12.77 to 14.15)	8.12 (5.73 to 10.52)	9.97 (8.85 to 11.08)	10.68 (9.49 to 11.86)	11.68 (10.51 to 12.84)	13.10 (11.99 to 14.21)	15.25 (13.20 to 17.29)	16.08 (13.77 to 18.39)	14.85 (12.82 to 16.88)	**4.11 (0.64 to 1.54)**	0.56 (-0.21 to 1.34)	**-3.55 (-4.44 to -2.65)**
Household income	Lowest and second quartile	19.01 (18.18 to 19.84)	11.64 (10.05 to 13.23)	14.66 (13.39 to 15.94)	14.61 (13.26 to 15.95)	17.62 (16.12 to 19.12)	19.51 (18.00 to 21.01)	23.25 (20.46 to 26.04)	24.57 (21.37 to 27.77)	20.41 (17.51 to 23.31)	**6.31 (1.32 to 2.33)**	0.41 (-0.65 to 1.47)	**-5.90 (-7.08 to -4.72)**
	Third and highest quartile	13.15 (12.47 to 13.83)	10.84 (9.01 to 12.67)	10.34 (9.26 to 11.42)	11.24 (10.02 to 12.45)	11.36 (10.15 to 12.56)	13.30 (12.11 to 14.48)	14.77 (12.54 to 17.00)	14.97 (12.69 to 17.25)	14.81 (12.62 to 16.99)	**2.99 (0.32 to 1.18)**	0.45 (-0.37 to 1.28)	**-2.54 (-3.47 to -1.61)**
Smoking status	Smoker or ex-smoker	17.36 (16.50 to 18.22)	13.28 (11.29 to 15.26)	13.28 (11.98 to 14.58)	14.26 (12.81 to 15.71)	15.39 (13.96 to 16.83)	16.92 (15.39 to 18.46)	19.84 (16.91 to 22.78)	22.25 (19.09 to 25.41)	18.60 (15.93 to 21.27)	**3.83 (0.54 to 1.60)**	0.70 (-0.34 to 1.74)	**-3.14 (-4.30 to -1.97)**
	Non-smoker	14.04 (13.36 to 14.72)	9.37 (7.86 to 10.87)	11.47 (10.37 to 12.57)	11.44 (10.21 to 12.67)	12.96 (11.75 to 14.17)	15.15 (13.93 to 16.38)	16.30 (14.12 to 18.49)	15.53 (12.92 to 18.14)	15.54 (13.28 to 17.81)	**5.01 (0.88 to 1.73)**	0.03 (-0.82 to 0.88)	**-4.98 (-5.93 to -4.02)**
*High central adiposity* ^a^													
Sex	Male	32.27 (28.81 to 35.73)	20.99 (10.90 to 31.08)	21.70 (15.35 to 28.05)	22.58 (15.46 to 29.71)	28.37 (21.89 to 34.84)	38.65 (32.38 to 44.91)	40.64 (31.18 to 50.09)	33.04 (22.95 to 43.13)	29.09 (20.82 to 37.35)	**14.65 (2.82 to 7.63)**	**-4.01 (-7.64 to -0.38)**	**-18.66 (-23.01 to -14.30)**
	Female	26.34 (24.50 to 28.19)	17.41 (12.96 to 21.85)	23.08 (19.70 to 26.46)	24.38 (20.64 to 28.12)	21.08 (17.72 to 24.44)	28.50 (24.97 to 32.03)	34.88 (29.21 to 40.55)	28.62 (22.88 to 34.35)	25.09 (18.72 to 31.46)	**5.35 (0.54 to 2.98)**	-1.77 (-4.14 to 0.61)	**-7.11 (-9.78 to -4.45)**
Age, years	30–39	16.65 (10.26 to 23.04)	2.45 (0.00 to 7.33)	7.93 (2.53 to 13.33)	4.92 (0.00 to 11.21)	12.67 (4.30 to 21.03)	13.37 (4.04 to 22.70)	24.91 (8.87 to 40.96)	25.74 (1.58 to 49.91)	20.26 (0.58 to 39.94)	**10.81 (-0.25 to 5.34)**	1.70 (-5.64 to 9.04)	**-9.11 (-16.97 to -1.25)**
	40–49	25.36 (20.20 to 30.52)	8.92 (0.00 to 18.57)	12.23 (4.98 to 19.49)	16.25 (7.97 to 24.54)	23.92 (13.78 to 34.05)	30.05 (19.86 to 40.25)	37.03 (20.67 to 53.39)	29.61 (13.70 to 45.52)	24.58 (10.11 to 39.04)	**18.33 (2.50 to 8.86)**	-2.98 (-9.12 to 3.15)	**-21.31 (-28.22 to -14.40)**
	50–59	30.19 (25.90 to 34.48)	18.53 (9.56 to 27.49)	23.65 (16.37 to 30.94)	22.66 (13.97 to 31.35)	16.77 (9.93 to 23.62)	31.90 (23.33 to 40.46)	38.46 (26.28 to 50.63)	35.91 (21.20 to 50.61)	34.08 (21.54 to 46.63)	6.08 (-0.83 to 4.79)	-0.02 (-5.25 to 5.21)	**-6.09 (-12.03 to -0.16)**
	≥60	30.02 (28.08 to 31.96)	22.05 (16.56 to 27.55)	27.52 (23.76 to 31.28)	29.30 (25.06 to 33.53)	26.33 (22.23 to 30.43)	34.32 (30.98 to 37.66)	38.51 (32.14 to 44.89)	29.36 (24.85 to 33.88)	25.78 (19.28 to 32.28)	**6.27 (0.90 to 3.49)**	**-3.62 (-6.02 to -1.22)**	**-9.89 (-12.62 to -7.16)**
Region of residence	Urban	28.36 (26.34 to 30.38)	16.02 (11.22 to 20.81)	23.53 (19.93 to 27.13)	25.44 (21.25 to 29.63)	22.34 (18.84 to 25.85)	30.89 (27.45 to 34.33)	37.64 (31.83 to 43.46)	30.51 (24.35 to 36.68)	25.95 (20.29 to 31.61)	**7.83 (1.35 to 3.86)**	**-2.49 (-4.74 to -0.24)**	**-10.32 (-12.90 to -7.75)**
	Rural	27.66 (24.78 to 30.54)	23.96 (16.27 to 31.66)	21.36 (15.79 to 26.92)	20.66 (15.11 to 26.21)	24.54 (18.35 to 30.73)	33.84 (27.43 to 40.25)	35.41 (24.46 to 46.36)	28.92 (20.59 to 37.24)	29.25 (20.20 to 38.31)	**8.78 (0.81 to 5.25)**	-2.12 (-5.85 to 1.62)	**-10.89 (-15.24 to -6.55)**
BMI group^b^	Underweight or normal weight	19.73 (1.93 to 37.52)	2.77 (0.00 to 8.50)	N/A	22.12 (0.00 to 60.52)	5.51 (0.00 to 16.36)	22.39 (0.00 to 46.39)	N/A	50.62 (0.00 to 100.00)	N/A	25.03 (-0.45 to 10.29)	12.70 (-14.20 to 39.59)	-12.33 (-39.76 to 15.09)
	Overweight	22.00 (15.74 to 28.26)	25.36 (9.59 to 41.13)	11.74 (4.85 to 18.62)	13.16 (3.81 to 22.50)	24.64 (9.47 to 39.80)	24.32 (10.51 to 38.14)	50.94 (22.51 to 79.38)	18.80 (5.16 to 32.45)	8.53 (0.00 to 24.06)	8.17 (-1.91 to 6.80)	**-8.40 (-15.68 to -1.12)**	**-16.57 (-25.05 to -8.09)**
	Obese	28.50 (26.79 to 30.22)	17.78 (13.54 to 22.02)	23.78 (20.57 to 27.00)	24.54 (21.02 to 28.05)	22.98 (19.84 to 26.11)	31.87 (28.68 to 35.05)	36.69 (31.48 to 41.91)	30.43 (25.11 to 35.74)	27.26 (22.43 to 32.09)	**7.79 (1.49 to 3.78)**	**-2.25 (-4.21 to -0.29)**	**-10.04 (-12.31 to -7.77)**
Level of education	Middle school or lower education	28.27 (26.34 to 30.20)	18.78 (14.32 to 23.23)	24.98 (21.19 to 28.78)	27.17 (23.14 to 31.19)	24.60 (20.66 to 28.54)	33.39 (29.57 to 37.21)	38.31 (31.12 to 45.50)	30.17 (24.51 to 35.82)	23.07 (16.12 to 30.02)	**7.94 (1.38 to 3.97)**	**-3.95 (-6.51 to -1.38)**	**-11.89 (-14.76 to -9.02)**
	College or higher education	28.07 (25.08 to 31.07)	12.58 (1.27 to 23.90)	16.66 (11.63 to 21.69)	17.39 (11.75 to 23.03)	20.06 (15.12 to 25.00)	29.02 (23.93 to 34.11)	36.14 (27.94 to 44.34)	30.09 (21.60 to 38.59)	29.40 (22.66 to 36.14)	**12.06 (2.09 to 6.31)**	-0.94 (-3.91 to 2.03)	**-13.00 (-16.64 to -9.36)**
Household income	Lowest and second quartile	29.74 (27.83 to 31.64)	21.49 (16.06 to 26.92)	26.21 (22.51 to 29.92)	26.53 (22.44 to 30.62)	24.77 (20.63 to 28.92)	33.09 (29.54 to 36.65)	36.31 (29.88 to 42.73)	31.86 (26.09 to 37.64)	29.28 (23.37 to 35.20)	**6.15 (0.80 to 3.45)**	-1.66 (-3.95 to 0.62)	**-7.81 (-10.45 to -5.17)**
	Third and highest quartile	25.83 (22.93 to 28.72)	11.31 (6.20 to 16.43)	15.96 (11.57 to 20.34)	18.83 (13.83 to 23.83)	19.63 (15.01 to 24.26)	28.75 (23.11 to 34.40)	38.21 (29.45 to 46.96)	27.81 (19.76 to 35.85)	23.65 (16.06 to 31.24)	**12.79 (2.18 to 5.79)**	**-3.30 (-6.57 to -0.02)**	**-16.08 (-19.82 to -12.34)**
Smoking status	Smoker or ex-smoker	31.54 (28.34 to 34.75)	18.57 (10.58 to 26.56)	20.74 (15.39 to 26.10)	25.04 (18.75 to 31.32)	28.29 (22.33 to 34.25)	37.39 (31.59 to 43.19)	41.62 (31.68 to 51.55)	33.77 (23.94 to 43.60)	28.78 (20.71 to 36.84)	**14.35 (2.90 to 7.03)**	**-3.81 (-7.32 to -0.31)**	**-18.17 (-22.24 to -14.10)**
	Non-smoker	26.54 (24.63 to 28.45)	17.79 (13.47 to 22.11)	23.64 (20.01 to 27.26)	23.71 (19.88 to 27.54)	20.56 (17.23 to 23.89)	28.91 (25.33 to 32.49)	34.78 (28.76 to 40.79)	28.20 (22.56 to 33.84)	25.31 (19.01 to 31.61)	**5.24 (0.47 to 2.99)**	-1.90 (-4.29 to 0.49)	**-7.14 (-9.84 to -4.44)**

Abbreviations: BMI, body mass index; CI, confidence interval; KNHANES, Korea National Health and Nutrition Examination Survey.

Beta values were multiplied by 100 due to their small magnitudes.

The values in bold font represent significant variance (*p* < 0.05).

^a^ According to the guidelines from the National Institute for Health and Care Excellence, central adiposity is divided into three groups: healthy central adiposity (waist-to-height ratio 0.40–0.49), increased central adiposity (waist-to-height ratio 0.50–0.59), and high central adiposity (waist-to-height ratio ≥ 0.60).

^b^ According to the Asian-Pacific guidelines, BMI is divided into four groups: underweight (< 18.5 kg/m^2^), normal (18.5–22.9 kg/m^2^), overweight (23.0–24.9 kg/m^2^), and obese (25.0–34.9 kg/m^2^).

Table 3 shows the factors associated with an increased risk of type 2 diabetes mellitus in each central adiposity group. Advanced age, lower educational attainment, lower household income, and current or former smoking status were identified as statistically significant risk factors for an increased likelihood of type 2 diabetes mellitus across all three groups. Furthermore, the risk of type 2 diabetes mellitus was higher in males than females in all groups. Compared with individuals in their 30s, individuals aged ≥ 60 years (ratio of ORs 17.72 [95% CI, 13.63 to 23.05]) exhibited a significantly higher risk of type 2 diabetes mellitus in the healthy central adiposity group. Furthermore, residing in rural areas and being overweight increased the risk of type 2 diabetes mellitus in the increased central adiposity group. Compared with those who were underweight or normal weight, individuals who were overweight (ratio of ORs 5.85 [95% CI, 2.54 to 13.47]) or obese (ratio of ORs 8.24 [95% CI, 3.79 to 17.94]) exhibited a significantly higher risk of type 2 diabetes mellitus in the high central adiposity group. However, when comparing the β values by dividing the entire year into pre-pandemic and pandemic periods, significant differences were observed among the three central groups. Otherwise, when analyzing the wOR for each period, the significant intervals varied, and no common significant period was identified (Table S3). The risk of type 2 diabetes mellitus tended to increase across all assessed variables; however, the individuals in the increased and high central adiposity groups were more susceptible to type 2 diabetes than the healthy central adiposity group (Table S4).

**Table 3:** Weighted odds ratios in the prevalence of type 2 diabetes mellitus, stratified by central adiposity groups before and during COVID–19 (weighted % [95% CI]).

Variables		Overall (2005–2022)		Before the pandemic (2005–2019)		During the pandemic (2020–2022)		Ratio of ORs (95% CI) during the pandemic compared to before the pandemic (reference)	
		Weighted OR (95% CI)	*P*-value	Weighted OR (95% CI)	*P*-value	Weighted OR (95% CI)	*P*-value	Weighted ratio of OR (95% CI)	*P*-value
*Healthy central adiposity* ^a^									
Sex	Female	1.00 (ref)		1.00 (ref)		1.00 (ref)		1.00 (ref)	
	Male	**2.19 (1.96 to 2.44)**	**< 0.001**	**2.31 (2.05 to 2.62)**	**< 0.001**	**1.87 (1.46 to 2.40)**	**< 0.001**	0.81 (0.61 to 1.07)	0.134
Age, years	30–39	1.00 (ref)		1.00 (ref)		1.00 (ref)		1.00 (ref)	
	40–49	**4.00 (3.01 to 5.32)**	**< 0.001**	**4.00 (2.98 to 5.35)**	**< 0.001**	**4.31 (1.39 to 13.35)**	**0.012**	1.08 (0.34 to 3.47)	0.900
	50–59	**9.51 (7.23 to 12.52)**	**< 0.001**	**8.45 (6.36 to 11.23)**	**< 0.001**	**18.63 (6.33 to 54.83)**	**< 0.001**	2.20 (0.72 to 6.73)	0.166
	≥60	**17.72 (13.63 to 23.05)**	**< 0.001**	**16.05 (12.27 to 21.00)**	**< 0.001**	**31.79 (10.94 to 92.37)**	**< 0.001**	1.98 (0.66 to 5.95)	0.223
Region of residence	Urban	1.00 (ref)		1.00 (ref)		1.00 (ref)		1.00 (ref)	
	Rural	**1.53 (1.34 to 1.75)**	**< 0.001**	**1.43 (1.23 to 1.67)**	**< 0.001**	**2.11 (1.57 to 2.84)**	**< 0.001**	**1.47 (1.06 to 2.05)**	**0.022**
BMI group^b^	Underweight or normal weight	1.00 (ref)		1.00 (ref)		1.00 (ref)		1.00 (ref)	
	Overweight	0.98 (0.85 to 1.12)	0.715	1.11 (0.95 to 1.29)	0.186	**0.53 (0.37 to 0.76)**	**0.001**	**0.48 (0.33 to 0.71)**	**< 0.001**
	Obese	0.84 (0.64 to 1.10)	0.207	0.90 (0.67 to 1.21)	0.501	0.62 (0.30 to 1.30)	0.207	0.69 (0.31 to 1.52)	0.357
Education level	College or higher education	1.00 (ref)		1.00 (ref)		1.00 (ref)		1.00 (ref)	
	Middle school or lower education	**2.17 (1.94 to 2.42)**	**< 0.001**	**2.19 (1.94 to 2.47)**	**< 0.001**	**2.57 (1.94 to 3.42)**	**< 0.001**	1.18 (0.86 to 1.60)	0.303
Household income	Third and highest quartile	1.00 (ref)		1.00 (ref)		1.00 (ref)		1.00 (ref)	
	Lowest and second quartile	**1.57 (1.41 to 1.74)**	**< 0.001**	**1.66 (1.48 to 1.87)**	**< 0.001**	**1.33 (1.03 to 1.71)**	**0.026**	0.80 (0.61 to 1.06)	0.114
Smoking status	Non-smoker	1.00 (ref)		1.00 (ref)		1.00 (ref)		1.00 (ref)	
	Smoker or ex-smoker	**2.97 (2.66 to 3.31)**	**< 0.001**	**3.15 (2.79 to 3.57)**	**< 0.001**	**2.42 (1.89 to 3.09)**	**< 0.001**	0.77 (0.58 to 1.01)	0.058
*Increased central adiposity* ^a^									
Sex	Female	1.00 (ref)		1.00 (ref)		1.00 (ref)		1.00 (ref)	
	Male	**1.20 (1.13 to 1.28)**	**< 0.001**	**1.18 (1.10 to 1.27)**	**< 0.001**	**1.18 (1.03 to 1.36)**	**0.015**	1.00 (0.86 to 1.17)	0.990
Age, years	30–39	1.00 (ref)		1.00 (ref)		1.00 (ref)		1.00 (ref)	
	40–49	**2.29 (1.91 to 2.75)**	**< 0.001**	**2.21 (1.80 to 2.71)**	**< 0.001**	**2.63 (1.72 to 4.01)**	**< 0.001**	1.19 (0.74 to 1.90)	0.472
	50–59	**4.10 (3.45 to 4.87)**	**< 0.001**	**3.86 (3.19 to 4.67)**	**< 0.001**	**5.00 (3.35 to 7.46)**	**< 0.001**	1.29 (0.83 to 2.02)	0.255
	≥60	**6.15 (5.21 to 7.25)**	**< 0.001**	**5.75 (4.79 to 6.91)**	**< 0.001**	**7.33 (5.00 to 10.75)**	**< 0.001**	1.28 (0.83 to 1.95)	0.262
Region of residence	Urban	1.00 (ref)		1.00 (ref)		1.00 (ref)		1.00 (ref)	
	Rural	**1.14 (1.05 to 1.24)**	**0.002**	**1.12 (1.01 to 1.23)**	**0.026**	**1.30 (1.11 to 1.53)**	**0.001**	1.17 (0.97 to 1.41)	0.103
BMI group^b^	Underweight or normal weight	1.00 (ref)		1.00 (ref)		1.00 (ref)		1.00 (ref)	
	Overweight	**1.18 (1.08 to 1.29)**	**< 0.001**	**1.30 (1.17 to 1.44)**	**< 0.001**	**0.80 (0.66 to 0.97)**	**0.021**	**0.62 (0.49 to 0.77)**	**< 0.001**
	Obese	1.03 (0.94 to 1.12)	0.551	**1.14 (1.04 to 1.26)**	**0.007**	**0.68 (0.57 to 0.81)**	**< 0.001**	**0.60 (0.49 to 0.73)**	**< 0.001**
Education level	College or higher education	1.00 (ref)		1.00 (ref)		1.00 (ref)		1.00 (ref)	
	Middle school or lower education	**1.73 (1.62 to 1.84)**	**< 0.001**	**1.75 (1.62 to 1.88)**	**< 0.001**	**2.12 (1.85 to 2.43)**	**< 0.001**	**1.21 (1.04 to 1.42)**	**0.014**
Household income	Third and highest quartile	1.00 (ref)		1.00 (ref)		1.00 (ref)		1.00 (ref)	
	Lowest and second quartile	**1.55 (1.45 to 1.64)**	**< 0.001**	**1.51 (1.41 to 1.62)**	**< 0.001**	**1.74 (1.54 to 1.98)**	**< 0.001**	1.15 (0.99 to 1.33)	0.051
Smoking status	Non-smoker	1.00 (ref)		1.00 (ref)		1.00 (ref)		1.00 (ref)	
	Smoker or ex-smoker	**1.44 (1.35 to 1.53)**	**< 0.001**	**1.41 (1.32 to 1.52)**	**< 0.001**	**1.48 (1.29 to 1.70)**	**< 0.001**	1.05 (0.90 to 1.22)	0.541
*High central adiposity* ^a^									
Sex	Female	1.00 (ref)		1.00 (ref)		1.00 (ref)		1.00 (ref)	
	Male	1.07 (0.94 to 1.23)	0.327	1.01 (0.86 to 1.19)	0.909	1.05 (0.81 to 1.35)	0.715	1.04 (0.77 to 1.40)	0.808
Age, years	30–39	1.00 (ref)		1.00 (ref)		1.00 (ref)		1.00 (ref)	
	40–49	**1.98 (1.39 to 2.80)**	**< 0.001**	**2.30 (1.52 to 3.47)**	**< 0.001**	1.61 (0.85 to 3.05)	0.143	0.70 (0.33 to 1.50)	0.360
	50–59	**2.32 (1.67 to 3.22)**	**< 0.001**	**2.65 (1.80 to 3.91)**	**< 0.001**	**2.00 (1.08 to 3.69)**	**0.027**	0.75 (0.37 to 1.56)	0.446
	≥60	**2.59 (1.93 to 3.46)**	**< 0.001**	**3.31 (2.36 to 4.66)**	**< 0.001**	1.70 (0.99 to 2.91)	0.055	**0.51 (0.27 to 0.97)**	**0.040**
Region of residence	Urban	1.00 (ref)		1.00 (ref)		1.00 (ref)		1.00 (ref)	
	Rural	0.93 (0.80 to 1.07)	0.292	0.95 (0.80 to 1.12)	0.525	0.93 (0.72 to 1.22)	0.608	0.99 (0.72 to 1.35)	0.925
BMI group^b^	Underweight or normal weight	1.00 (ref)		1.00 (ref)		1.00 (ref)		1.00 (ref)	
	Overweight	**5.85 (2.54 to 13.47)**	**< 0.001**	**10.66 (4.19 to 27.12)**	**< 0.001**	0.55 (0.10 to 2.86)	0.472	**0.05 (0.01 to 0.34)**	**0.002**
	Obese	**8.24 (3.79 to 17.94)**	**< 0.001**	**16.00 (6.72 to 38.08)**	**< 0.001**	0.59 (0.13 to 2.77)	0.503	**0.04 (0.01 to 0.22)**	**< 0.001**
Education level	College or higher education	1.00 (ref)		1.00 (ref)		1.00 (ref)		1.00 (ref)	
	Middle school or lower education	**1.26 (1.10 to 1.45)**	**0.001**	**1.43 (1.21 to 1.68)**	**< 0.001**	1.12 (0.87 to 1.45)	0.373	0.79 (0.58 to 1.07)	0.127
Household income	Third and highest quartile	1.00 (ref)		1.00 (ref)		1.00 (ref)		1.00 (ref)	
	Lowest and second quartile	**1.35 (1.18 to 1.54)**	**< 0.001**	**1.49 (1.27 to 1.75)**	**< 0.001**	1.19 (0.93 to 1.52)	0.160	0.80 (0.60 to 1.07)	0.132
Smoking status	Non-smoker	1.00 (ref)		1.00 (ref)		1.00 (ref)		1.00 (ref)	
	Smoker or ex-smoker	**1.32 (1.16 to 1.51)**	**< 0.001**	**1.28 (1.09 to 1.50)**	**0.002**	**1.34 (1.04 to 1.73)**	**0.026**	1.05 (0.78 to 1.42)	0.756

Abbreviations: BMI, body mass index; CI, confidence interval; KNHANES, Korea National Health and Nutrition Examination Survey; OR, odds ratio.

The values in bold font represent a significant variance (*p* < 0.05).

^a^ According to the guidelines from the National Institute for Health and Care Excellence, central adiposity is divided into three groups: healthy central adiposity (waist-to-height ratio 0.40–0.49), increased central adiposity (waist-to-height ratio 0.50–0.59), and high central adiposity (waist-to-height ratio ≥ 0.60).

^b^ According to the Asian-Pacific guidelines, BMI is divided into four groups: underweight (< 18.5 kg/m^2^), normal (18.5–22.9 kg/m^2^), overweight (23.0–24.9 kg/m^2^), and obese (25.0–34.9 kg/m^2^).

## Discussion

### Key findings

This study used KNHANES data for an 18-year period spanning from 2005 to 2022 to analyze the trend of type 2 diabetes mellitus by stratifying central adiposity into the healthy, increased, and high central adiposity group (*n* = 79,368). The prevalence of type 2 diabetes mellitus increased from 3.32 to 5.80%, 11.22–17.07%, and 18.00–26.70% in the healthy, increased, and high central adiposity group, respectively. The risk of developing type 2 diabetes mellitus was consistently associated with the following socio-demographic factors: older population (≥ 60 years), middle school or lower education level, lower household income, and smoking. In the healthy central adiposity group, susceptibility to developing type 2 diabetes mellitus increased significantly with age. Therefore, various factors, particularly aging, increase the risk of developing type 2 diabetes mellitus, even in the healthy central obesity groups. In addition, the risk of developing type 2 diabetes mellitus was higher in males than in females. Furthermore, only in individuals with high central adiposity was an increased risk of developing type 2 diabetes mellitus with higher BMI observed; this association was inconsistent in other groups.

## Comparison with previous studies

Compared to previous studies, our findings corroborate existing research that indicates an increasing prevalence of type 2 diabetes mellitus across various socio-demographic groups and levels of central adiposity. The role of reduced physical activity, unhealthy dietary habits, and socioeconomic factors in exacerbating the risk of developing type 2 diabetes has been consistently emphasized in previous studies^24,25^. These factors contribute to the development of insulin resistance and metabolic dysfunction, which are central to the development of diabetes.

Several recent studies have highlighted significant sex differences in the risk of developing diabetes. For instance, research has consistently shown that males exhibit higher rates of abdominal obesity and associated insulin resistance compared to females^26,27^. This aligns with our findings, which reveal that males are more susceptible to developing type 2 diabetes mellitus across all levels of central obesity. Furthermore, the literature underscores a progressive increase in diabetes risk with aging^28–31^. Our study corroborates these findings and emphasizes the heightened vulnerability of older adults, including those who initially have healthy central adiposity. The findings of the present study confirm these trends and emphasize the significant vulnerability of older adults, even those with initially healthy central adiposity.

In the context of obesity and diabetes mellitus risk, obesity is closely linked to a high prevalence of type 2 diabetes and its complications^32^. Various anthropometric methods and surrogate adipose indices are used to assess the risk of diabetes and insulin resistance^33^. BMI is one of the most commonly used clinical measures for determining obesity. However, previous studies have found that the WHtR correlates significantly better with body fat than BMI^34,35^.

Our study supports existing findings regarding the WHtR. When evaluating and analyzing central fat using WHtR, a significant increase in the risk of type 2 diabetes was observed only in patients with high central obesity across all BMI categories. While both BMI and WHtR can be utilized to assess diabetes prevalence based on levels of obesity, our results suggest that WHtR is more sensitive in evaluating obesity and more effectively captures diabetes prevalence. This underscores the importance of using both WHtR and BMI together to understand the impact of central obesity on the prevalence of type 2 diabetes.

Similar patterns have emerged internationally. In Japan, the rising prevalence of type 2 diabetes has been accompanied by an increase in central obesity, despite BMI levels remaining relatively stable, reinforcing the stronger association between abdominal obesity and diabetes risk^36^. In China, WHtR has proven more effective than BMI and waist-to-hip ratio (WHR) in predicting cardiovascular risk in patients with diabetes^37^. Furthermore, research from the UK indicates that WHtR is not only a more effective indicator than BMI for diabetes risk but also a simpler and more reliable metric for identifying a range of other health conditions^38^.

Additionally, we compared the trends of type 2 diabetes in Korea with other countries. Globally, the prevalence of type 2 diabetes has been increasing due to factors such as rising rates of overweight individuals and shifts in dietary habits^6,39,40^. Countries such as the UK, Ireland, and Luxembourg had reported a more than 1.2 fold increase in diabetes prevalence by 2019 compared to 1990^41^. Similarly, in the U.S., the prevalence of type 2 diabetes increased from 9.8% during 1999–2000 to 14.3% by 2017–2018, primarily attributed to a substantial rise in obesity rates and widening socioeconomic disparities^42,43^. Likewise, our study showed an increase in the prevalence of type 2 diabetes in South Korea, from 8.74% in 2005–2007 to 15.19% in 2022.

## Plausible underlying mechanisms

In our study, we observed an increasing trend in the prevalence of type 2 diabetes mellitus was observed in all central adiposity groups with significantly higher prevalence observed in 2022 compared to 2005. This increase could be attributed to lifestyle changes over the past decades, including increased consumption of high-calorie, processed foods, which contributed to weight gain and central adiposity^44,45^. Additionally, the aging population and prolonged exposure to risk factors such as sedentary behavior and unhealthy dietary habits likely played a significant role in the rising prevalence of type 2 diabetes mellitus^46^.

This study showed that several socio-demographic increased susceptibility to type 2 diabetes across all central adiposity groups. Individuals with lower education levels and household incomes may possess limited health knowledge and access to healthcare, further exacerbating their risk of developing type 2 diabetes mellitus^47^. These groups may have encounter barriers in obtaining the information and resources necessary for the prevention and management of the disease, hindering early detection and appropriate treatment of type 2 diabetes mellitus. Nicotine, a major physiologically active component of tobacco, may disrupt glucose homeostasis and lead to abnormalities in glucose metabolism^48,49^. Moreover, males exhibit a higher vulnerability of developing type 2 diabetes mellitus compared to females across all levels of central adiposity. Females are more likely to accumulate adipose tissue in the hips and thighs, while males tend to accumulate fat in the abdominal region. Abdominal obesity is associated with insulin resistance, which is generally higher in males than in females^50^.

We found that the risk of developing type 2 diabetes mellitus increased with age in all adiposity groups. Specifically, in our study, the healthy central adiposity group showed a significant risk of type 2 diabetes among the older population (≥ 60 years) compared to those in their 30s. This finding suggests that the risk of developing type 2 diabetes mellitus increases significantly in the 60s due to aging, even in individuals with healthy central obesity^51^. Furthermore, the findings of this study suggest that the individuals in the increased central adiposity group are likely to develop diabetes at an earlier age than those in the healthy central adiposity group but still later than those with high central adiposity. Individuals with high central adiposity may develop diabetes much earlier. This progression indicates that the onset of type 2 diabetes mellitus occurs earlier as the central adiposity levels increase^52^. Factors such as the decline in metabolic functions, increased insulin resistance, decreased physical activity, hormonal changes, and chronic inflammation collectively heighten the risk of developing type 2 diabetes in aging individuals. Therefore, individuals with high central adiposity are significantly more susceptible to developing early-onset diabetes than those with increased or healthy central adiposity.

Interestingly, this study found that an increased risk of developing type 2 diabetes mellitus associated with higher BMI was only observed in the high central adiposity group. While BMI is commonly used to measure overall body fat, it may have limitations in accurately reflecting central adiposity, as it does not account for the distribution of fat^53^. This is particularly relevant, given that central adiposity—defined as fat accumulation in the abdominal area— has been associated with an increased risk of metabolic disorders, such as type 2 diabetes and cardiovascular disease. In contrast, WHtR offers an additional assessment of central adiposity by considering waist circumference in relation to height. Research indicates that WHtR may be a more effective predictor of health risks associated with obesity, especially those linked to abdominal fat accumulation^34,54^. Therefore, this study suggests that using both BMI and WHtR in combination could improve the identification of individuals at higher risk for metabolic complications, thereby supporting more targeted prevention and management strategies.

### Clinical and policy implications

The present study highlights the need for increased screening and monitoring of type 2 diabetes mellitus among individuals with high central adiposity. Healthcare professionals should use WHtR alongside BMI for a more precise risk assessment. Regular monitoring and targeted screening may facilitate early detection and intervention. Personalized lifestyle modifications are crucial for the older population (≥ 60 years) with high central adiposity. Enhancing health education and support systems is vital for effective diabetes risk management. Public health campaigns that promote physical activity and healthy eating habits, particularly among low-income populations, are essential for disease prevention. Furthermore, policy measures should aim to improve access to healthcare and incorporate WHtR into clinical guidelines to enhance health outcomes.

### Strength and limitations

This study has several limitations. First, as a cross-sectional study, it does not allow for tracking changes within the same individuals, which limits our ability to understand how factors such as aging, lifestyle changes, or urbanization may influence the development or progression of diabetes over time. For example, although we analyzed prevalence trends before and after the COVID-19 pandemic, we were unable to assess how individual risk factors evolved or contributed to changes in diabetes prevalence^55–57^. To better understand these dynamic relationships, future studies employing longitudinal data would be necessary to elucidate the temporal effects of urbanization and other evolving factors on type 2 diabetes mellitus risk^58^, stratified by WHtR. Second, the study focused on individuals aged 30 and above, aligning with the standard age range for adult type 2 diabetes mellitus in many existing studies. Consequently, the 0–29 years age group was excluded, which omitted information regarding changes in the prevalence of type 2 diabetes mellitus among younger individuals and related prevention strategies. While the KNHANES dataset does not distinguish between diabetes types, analyzing individuals aged 30 and older aligns with prior studies for targeting type 2 diabetes^59,60^. However, this focus facilitated a more precise analysis of adults with type 2 diabetes mellitus. Third, type 2 diabetes mellitus is influenced by various lifestyle and environmental factors. Due to a lack of data, we were unable to account for all relevant factors; however, this analysis included other lifestyle-related factors such as BMI and smoking. Additionally, the dataset utilized in the study is unable to classify the specific types of diabetes mellitus. This presents a potential limitation in the approach, as individuals with other forms of diabetes mellitus may be misclassified as having type 2 diabetes. Although this issue may only affect a small subset of cases, it nonetheless restricts the accuracy of the classification of type 2 diabetes cases. Lastly, the use of self-reported data introduces potential recall and social desirability biases. Recall bias may lead to inaccuracies, while social desirability bias may compel respondents to provide answers they perceive as more acceptable to others rather than reflecting their true thoughts or behaviors^56^.

Despite these limitations, the strength of this study lies in the use of nationwide representative data spanning 18 years to monitor the prevalence trends of type 2 diabetes mellitus, stratified by level of central adiposity. We present representative trends for South Korea based on comprehensive nationwide data collected over an extended period. Furthermore, we examined the sociodemographic factors and their association with central obesity in patients with type 2 diabetes mellitus, identifying older age, low education level, low household income, and smoking status as significant risk factors. Finally, this study emphasizes the importance of identifying and managing risk factors related to central obesity in patients with type 2 diabetes mellitus.

## Conclusion

This study identified an increasing prevalence of type 2 diabetes mellitus in all central adiposity groups from 2005 to 2022, including during the COVID-19 pandemic. Specifically, the prevalence of type 2 diabetes mellitus rose from 3.32 to 5.80% in the healthy central adiposity group, from 11.22 to 17.07% in the increased central adiposity group, and from 18.00 to 26.70% in the high central adiposity group over this period. Our study found that socio-demographic factors such as older age (≥ 60 years), lower education levels, lower household income, and smoking universally increase the risk of developing type 2 diabetes mellitus, with males being more susceptible than females, regardless of central adiposity levels. This study contributes to developing more effective diabetes screening and management strategies by emphasizing the importance of using WHtR alongside BMI for risk assessment and advocating for targeted interventions and policy measures to improve health outcomes among individuals with high central adiposity.

## Electronic supplementary material

Below is the link to the electronic supplementary material.

Supplementary Material 1

## Data Availability

The data are available upon request. Study protocol and statistical codes: Available from DKY (yonkkang@gmail.com). Dataset: Available from the Korea Disease Control and Prevention Agency through a data use agreement. Korea National Health and Nutrition Examination Survey is public available as followed link: https://knhanes.kdca.go.kr/knhanes/sub03/sub03_02_05.do.
