# Supplementary material for: National trends in type 2 diabetes mellitus stratified by central adiposity using waist-to-height ratio in South Korea, 2005–2022
Source: Sci Rep. 2024 Oct 16;14:24273. doi: 10.1038/s41598-024-75002-2 (PMC11484852; doi:10.1038/s41598-024-75002-2)
Supplement: Supplementary file 1 — Supplementary Material 1 [file 41598_2024_75002_MOESM1_ESM.docx]

| **Supplementary Material** |
| --- |

Original Article

National trends in type 2 diabetes mellitus stratified by central adiposity using waist-to-height ratio in South Korea, 2005–2022

Running title: Trends in type 2 diabetes mellitus according to central adiposity

Hyunjee Kim^1,2†^, Seoyoung Park^1,2†^, Jaeyu Park^1,3^, Yejun Son^1,2^, Soeun Kim^1,2^, Yesol Yim^1,2^, Hyesu Jo^1,3^, Kyeongmin Lee^1,3^, Yi Deun Jeong^1,4^, Jiyeon Oh^1,4^, Hanseul Cho^1,4^, Damiano Pizzol^5,6^, Jiyoung Hwang^1*^, Lee Smith^7*^, Dong Keon Yon^1,2,3,4,8*^

^†^ These authors contributed equally to this work as first authors.

* These authors contributed equally to this work as corresponding authors.

*Corresponding authors:

**Dong Keon Yon**, MD, PhD, FACAAI, FAAAAI (lead contact)

Department of Pediatrics, Kyung Hee University College of Medicine, 23 Kyungheedae–ro, Dongdaemun–gu, Seoul 02447, South Korea

Tel: +82–2–6935–2476

Fax: +82–504–478–0201

Email: yonkkang@gmail.com

**Contents of supplementary appendix**

| **Supplementary Material** | | **Page** |
| --- | --- | --- |
| **Figure S1** | Study population in KNHANES, 2005–2022. | P3 |
| **Table S1** | National trends in the prevalence of type 2 diabetes mellitus among Korean adults, 2005–2022 | P4–8 |
| **Table S2** | National trends in the prevalence of type 2 diabetes mellitus, stratified by central adiposity groups and β-coefficients of odds ratios comparing before and during the COVID–19 pandemic (weighted % [95% CI]). | P9–16 |
| **Table S3** | Weighted odds ratios in the periods for prevalence of type 2 diabetes mellitus, stratified by central adiposity groups (weighted % [95% CI]) | P17–23 |
| **Table S4** | Weighted odds ratios in the prevalence of type 2 diabetes mellitus, stratified by central adiposity groups, before and during the COVID–19 pandemic (weighted % [95% CI]) | P24–28 |

**Figure S1.** Study population in KNHANES, 2005–2022.

**
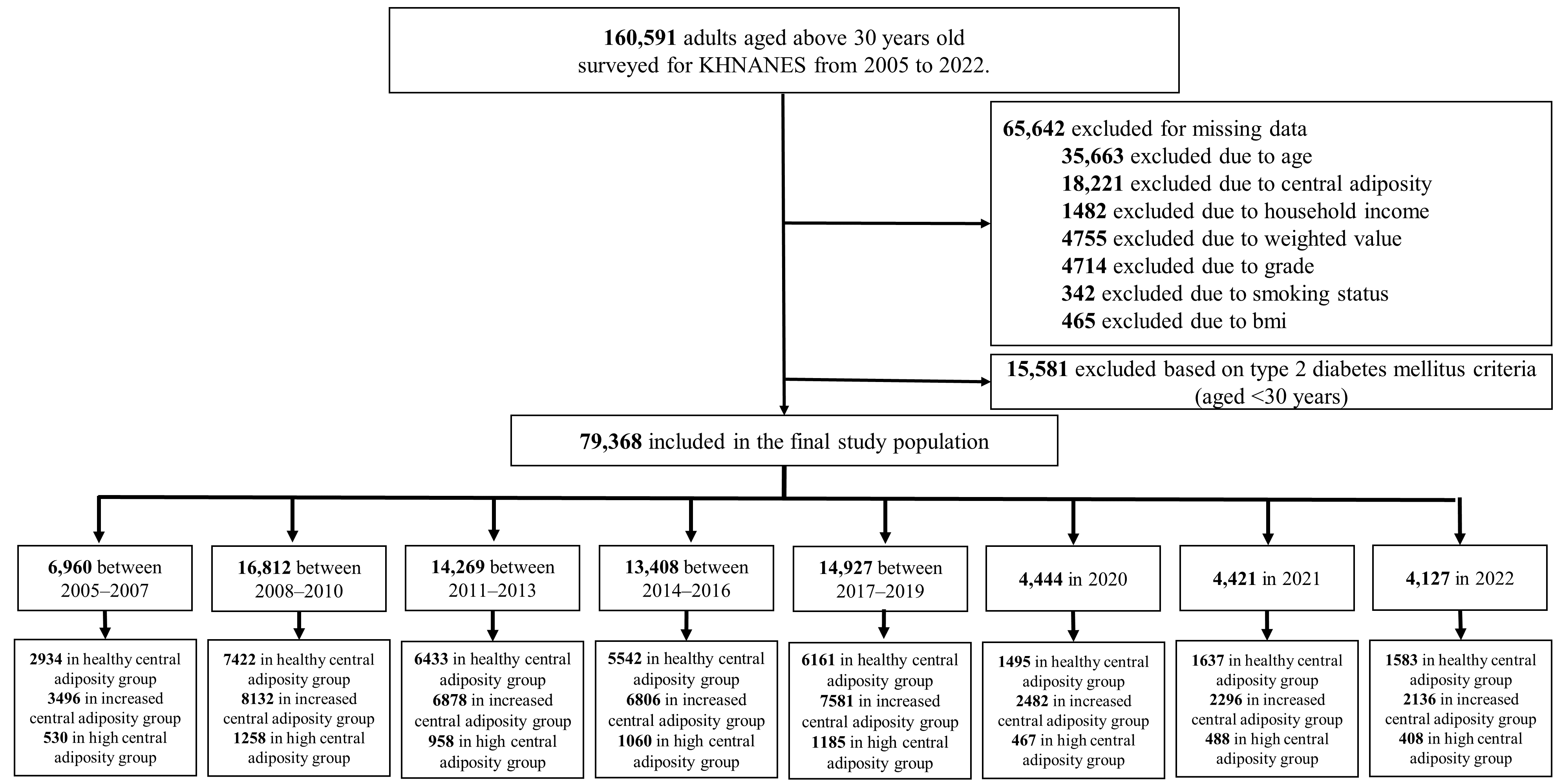
**

Abbreviations: KNHANES, Korea National Health and Nutrition Examination Survey.

**Table S1.** National trends in the prevalence of type 2 diabetes mellitus among Korean adults, 2005–2022.

| Variables | Total | Pre-pandemic | | | | | During the pandemic | | |
| --- | --- | --- | --- | --- | --- | --- | --- | --- | --- |
|  |  | 2005–2007 | 2008–2010 | 2011–2013 | 2014–2016 | 2017–2019 | 2020 | 2021 | 2022 |
| **Crude rate, n (%)** |  |  |  |  |  |  |  |  |  |
| **Overall, n (%)** | 9,920 (12.50) | 608 (8.74) | 1,744 (10.37) | 1,615 (11.32) | 1,666 (12.43) | 2,101 (14.08) | 779 (17.53) | 780 (17.64) | 627 (15.19) |
| **Sex, n (%)** |  |  |  |  |  |  |  |  |  |
| Male | 5,128 (14.99) | 317 (10.76) | 862 (11.94) | 823 (13.65) | 869 (15.20) | 1,114 (17.04) | 401 (20.17) | 406 (20.74) | 336 (18.57) |
| Female | 4,792 (10.61) | 291 (7.25) | 882 (9.20) | 792 (9.61) | 797 (10.36) | 987 (11.77) | 378 (15.39) | 374 (15.18) | 291 (12.55) |
| **Age, years, n (%)** |  |  |  |  |  |  |  |  |  |
| 30–39 | 337 (2.27) | 29 (1.70) | 82 (2.20) | 53 (1.95) | 61 (2.51) | 54 (2.24) | 27 (4.00) | 16 (2.88) | 15 (2.53) |
| 40–49 | 1,058 (6.29) | 97 (5.41) | 215 (5.67) | 165 (5.66) | 175 (6.46) | 211 (6.85) | 67 (7.58) | 86 (9.80) | 42 (5.36) |
| 50–59 | 2,111 (12.37) | 159 (11.71) | 393 (11.44) | 352 (11.12) | 322 (10.80) | 428 (12.85) | 182 (18.27) | 133 (14.18) | 142 (16.53) |
| ≥60 | 6,414 (20.93) | 323 (15.40) | 1,054 (18.00) | 1,045 (19.12) | 1,108 (20.95) | 1,408 (23.07) | 503 (26.63) | 545 (26.59) | 428 (22.62) |
| **Region of residence, n (%)** |  |  |  |  |  |  |  |  |  |
| Urban | 7,326 (11.94) | 437 (8.57) | 1,236 (10.05) | 1,220 (10.90) | 1,285 (12.01) | 1,570 (13.09) | 588 (16.78) | 552 (16.38) | 438 (13.75) |
| Rural | 2,594 (14.39) | 171 (9.19) | 508 (11.24) | 395 (12.85) | 381 (14.06) | 531 (18.09) | 191 (20.32) | 228 (21.71) | 189 (20.06) |
| **Central adiposity ^a^, n (%)** |  |  |  |  |  |  |  |  |  |
| Healthy central adiposity | 1,905 (5.74) | 110 (3.75) | 367 (4.94) | 382 (5.94) | 320 (5.77) | 378 (6.14) | 100 (6.69) | 136 (8.31) | 112 (7.08) |
| Increased central adiposity | 6,304 (15.84) | 402 (11.50) | 1,088 (13.38) | 987 (14.35) | 1,099 (16.15) | 1,336 (17.62) | 504 (20.31) | 487 (21.21) | 401 (18.77) |
| High central adiposity | 1,711 (26.93) | 96 (18.11) | 289 (22.97) | 246 (25.68) | 247 (23.30) | 387 (32.66) | 175 (37.47) | 157 (32.17) | 114 (27.94) |
| **BMI group ^b^, n (%)** |  |  |  |  |  |  |  |  |  |
| Underweight or normal weight | 2,668 (8.49) | 144 (5.29) | 481 (7.01) | 456 (7.91) | 450 (8.49) | 550 (9.34) | 196 (12.05) | 204 (12.33) | 187 (11.53) |
| Overweight | 2,398 (12.08) | 180 (9.81) | 416 (9.78) | 398 (11.00) | 419 (12.50) | 492 (13.42) | 184 (17.13) | 176 (16.25) | 133 (13.57) |
| Obese | 4,854 (17.29) | 284 (11.82) | 847 (14.87) | 761 (15.58) | 797 (16.75) | 1,059 (19.71) | 399 (22.88) | 400 (23.75) | 307 (20.13) |
| **Level of education, n (%)** |  |  |  |  |  |  |  |  |  |
| Middle school or lower education | 5,483 (17.31) | 526 (9.53) | 1,096 (15.05) | 921 (16.44) | 884 (18.45) | 1,075 (22.72) | 343 (27.07) | 365 (27.08) | 273 (24.05) |
| College or higher education | 4,437 (9.30) | 82 (5.68) | 648 (6.80) | 694 (8.01) | 782 (9.08) | 1,026 (10.06) | 436 (13.72) | 415 (13.50) | 354 (11.83) |
| **Household income, n (%)** |  |  |  |  |  |  |  |  |  |
| Lowest and second quartile | 5,903 (16.14) | 359 (10.56) | 1,074 (13.50) | 960 (14.24) | 1,014 (16.69) | 1,267 (18.84) | 410 (21.77) | 465 (23.79) | 354 (19.31) |
| Third and highest quartile | 4,017 (9.38) | 249 (6.99) | 670 (7.56) | 655 (8.70) | 652 (8.89) | 834 (10.17) | 369 (14.41) | 315 (12.77) | 273 (11.90) |
| **Smoking status, n (%)** |  |  |  |  |  |  |  |  |  |
| Smoker or ex-smoker | 4,771 (14.70) | 300 (10.46) | 819 (11.63) | 787 (13.56) | 807 (15.12) | 1,008 (16.54) | 359 (19.70) | 379 (21.10) | 312 (18.46) |
| Non-smoker | 5,149 (10.97) | 308 (7.53) | 925 (9.47) | 828 (9.78) | 859 (10.64) | 1,093 (12.37) | 420 (16.02) | 401 (15.28) | 315 (12.93) |
| **Weighted rate (95% CI)** |  |  |  |  |  |  |  |  |  |
| **Overall, weighted % (95% CI)** | 11.25 (10.98 to 11.53) | 0.56 (0.49 to 0.64) | 1.68 (1.54 to 1.81) | 1.77 (1.61 to 1.93) | 1.93 (1.77 to 2.09) | 2.45 (2.26 to 2.65) | 0.93 (0.79 to 1.08) | 1.02 (0.86 to 1.17) | 0.91 (0.82 to 0.99) |
| **Sex, weighted % (95% CI)** |  |  |  |  |  |  |  |  |  |
| Male | 13.00 (12.58 to 13.42) | 0.66 (0.55 to 0.77) | 1.85 (1.67 to 2.03) | 2.03 (1.81 to 2.24) | 2.21 (2.00 to 2.43) | 2.86 (2.59 to 3.12) | 1.09 (0.90 to 1.28) | 1.23 (1.02 to 1.44) | 1.07 (0.94 to 1.20) |
| Female | 9.57 (9.23 to 9.90) | 0.47 (0.40 to 0.55) | 1.51 (1.36 to 1.66) | 1.52 (1.35 to 1.70) | 1.65 (1.48 to 1.82) | 2.07 (1.86 to 2.27) | 0.78 (0.63 to 0.92) | 0.81 (0.67 to 0.96) | 0.75 (0.65 to 0.86) |
| **Age, years, weighted % (95% CI)** |  |  |  |  |  |  |  |  |  |
| 30–39 | 2.41 (2.11 to 2.71) | 0.19 (0.10 to 0.28) | 0.46 (0.34 to 0.57) | 0.42 (0.29 to 0.55) | 0.44 (0.30 to 0.58) | 0.40 (0.28 to 0.52) | 0.20 (0.11 to 0.29) | 0.16 (0.06 to 0.26) | 0.15 (0.06 to 0.24) |
| 40–49 | 6.71 (6.24 to 7.17) | 0.43 (0.34 to 0.52) | 1.22 (1.02 to 1.43) | 1.09 (0.88 to 1.30) | 1.25 (1.03 to 1.47) | 1.33 (1.08 to 1.57) | 0.48 (0.33 to 0.62) | 0.60 (0.44 to 0.76) | 0.31 (0.20 to 0.41) |
| 50–59 | 13.12 (12.52 to 13.72) | 0.69 (0.55 to 0.82) | 1.98 (1.73 to 2.24) | 2.16 (1.87 to 2.46) | 2.10 (1.82 to 2.38) | 2.78 (2.44 to 3.12) | 1.19 (0.92 to 1.45) | 1.03 (0.80 to 1.27) | 1.19 (0.95 to 1.43) |
| ≥60 | 21.06 (20.51 to 21.61) | 0.88 (0.74 to 1.03) | 2.84 (2.57 to 3.10) | 3.18 (2.85 to 3.51) | 3.61 (3.27 to 3.96) | 4.89 (4.46 to 5.33) | 1.73 (1.43 to 2.03) | 2.09 (1.75 to 2.42) | 1.84 (1.63 to 2.05) |
| **Region of residence, weighted % (95% CI)** |  |  |  |  |  |  |  |  |  |
| Urban | 10.82 (10.51 to 11.12) | 0.53 (0.44 to 0.62) | 1.58 (1.43 to 1.73) | 1.64 (1.47 to 1.81) | 1.89 (1.71 to 2.07) | 2.38 (2.16 to 2.59) | 0.94 (0.77 to 1.11) | 0.97 (0.80 to 1.14) | 0.89 (0.77 to 1.00) |
| Rural | 13.12 (12.42 to 13.82) | 0.71 (0.55 to 0.88) | 2.10 (1.71 to 2.50) | 2.35 (1.83 to 2.86) | 2.09 (1.62 to 2.56) | 2.78 (2.20 to 3.37) | 0.89 (0.57 to 1.21) | 1.21 (0.78 to 1.64) | 0.99 (0.68 to 1.29) |
| **Central adiposity ^a^, weighted % (95% CI)** |  |  |  |  |  |  |  |  |  |
| Healthy central adiposity | 5.25 (4.97 to 5.53) | 0.23 (0.17 to 0.28) | 0.86 (0.75 to 0.98) | 1.07 (0.91 to 1.23) | 0.93 (0.80 to 1.07) | 1.08 (0.94 to 1.22) | 0.29 (0.22 to 0.37) | 0.43 (0.33 to 0.53) | 0.35 (0.28 to 0.43) |
| Increased central adiposity | 14.58 (14.16 to 15.00) | 0.78 (0.65 to 0.90) | 2.14 (1.94 to 2.33) | 2.17 (1.95 to 2.38) | 2.54 (2.30 to 2.77) | 3.19 (2.91 to 3.47) | 1.24 (1.03 to 1.45) | 1.33 (1.10 to 1.56) | 1.21 (1.07 to 1.34) |
| High central adiposity | 26.45 (25.14 to 27.77) | 1.24 (0.91 to 1.57) | 3.71 (3.09 to 4.32) | 3.52 (2.89 to 4.15) | 4.00 (3.33 to 4.67) | 6.14 (5.30 to 6.97) | 2.85 (2.24 to 3.47) | 2.59 (1.97 to 3.22) | 2.40 (1.91 to 2.90) |
| **BMI group ^b^, weighted % (95% CI)** |  |  |  |  |  |  |  |  |  |
| Underweight and normal weight | 7.54 (7.20 to 7.88) | 0.31 (0.25 to 0.37) | 1.12 (0.98 to 1.27) | 1.34 (1.15 to 1.52) | 1.27 (1.11 to 1.43) | 1.62 (1.44 to 1.80) | 0.54 (0.42 to 0.65) | 0.69 (0.55 to 0.82) | 0.65 (0.54 to 0.77) |
| Overweight | 10.54 (10.05 to 11.04) | 0.66 (0.54 to 0.79) | 1.59 (1.39 to 1.79) | 1.69 (1.46 to 1.93) | 1.87 (1.63 to 2.10) | 2.25 (1.97 to 2.53) | 0.91 (0.71 to 1.11) | 0.81 (0.63 to 0.98) | 0.77 (0.62 to 0.92) |
| Obese | 15.68 (15.17 to 16.19) | 0.76 (0.62 to 0.91) | 2.33 (2.10 to 2.56) | 2.29 (2.04 to 2.54) | 2.66 (2.39 to 2.94) | 3.48 (3.16 to 3.80) | 1.37 (1.12 to 1.61) | 1.51 (1.25 to 1.78) | 1.27 (1.11 to 1.44) |
| **Level of education, weighted % (95% CI)** |  |  |  |  |  |  |  |  |  |
| Middle school or lower education | 16.89 (16.39 to 17.39) | 1.44 (1.25 to 1.63) | 3.03 (2.74 to 3.32) | 2.90 (2.59 to 3.22) | 2.84 (2.54 to 3.13) | 3.46 (3.12 to 3.81) | 1.08 (0.87 to 1.29) | 1.17 (0.96 to 1.39) | 0.96 (0.81 to 1.10) |
| College or higher education | 8.69 (8.39 to 9.00) | 0.16 (0.12 to 0.21) | 1.07 (0.95 to 1.19) | 1.26 (1.12 to 1.40) | 1.51 (1.36 to 1.67) | 2.00 (1.81 to 2.19) | 0.86 (0.71 to 1.01) | 0.95 (0.78 to 1.11) | 0.89 (0.77 to 1.00) |
| **Household income, weighted % (95% CI)** |  |  |  |  |  |  |  |  |  |
| Lowest and second quartile | 14.73 (14.31 to 15.16) | 0.73 (0.61 to 0.86) | 2.35 (2.13 to 2.58) | 2.38 (2.13 to 2.63) | 2.60 (2.34 to 2.85) | 3.24 (2.94 to 3.55) | 1.07 (0.87 to 1.27) | 1.28 (1.06 to 1.51) | 1.07 (0.93 to 1.21) |
| Third and highest quartile | 8.81 (8.49 to 9.13) | 0.44 (0.37 to 0.52) | 1.21 (1.07 to 1.34) | 1.34 (1.19 to 1.50) | 1.46 (1.30 to 1.62) | 1.90 (1.71 to 2.09) | 0.83 (0.68 to 0.99) | 0.83 (0.68 to 0.99) | 0.79 (0.68 to 0.90) |
| **Smoking status, weighted % (95% CI)** |  |  |  |  |  |  |  |  |  |
| Smoker or ex-smoker | 12.85 (12.43 to 13.28) | 0.67 (0.56 to 0.78) | 1.88 (1.69 to 2.07) | 2.07 (1.85 to 2.30) | 2.21 (1.99 to 2.43) | 2.72 (2.46 to 2.98) | 1.04 (0.86 to 1.22) | 1.22 (1.00 to 1.43) | 1.05 (0.93 to 1.18) |
| Non-smoker | 9.92 (9.58 to 10.25) | 0.48 (0.40 to 0.55) | 1.51 (1.37 to 1.66) | 1.52 (1.35 to 1.69) | 1.69 (1.52 to 1.86) | 2.23 (2.02 to 2.45) | 0.84 (0.69 to 0.99) | 0.85 (0.70 to 1.00) | 0.79 (0.69 to 0.89) |

Abbreviations: BMI, body mass index; CI, confidence interval; KNHANES, Korea National Health and Nutrition Examination Survey.

^a^ According to guidelines from the National Institute for Health and Care Excellence, central adiposity is divided into three groups: healthy central adiposity (waist-to-height ratio 0.40–0.49), increased central adiposity (waist-to-height ratio 0.50–0.59), and high central adiposity (waist-to-height ratio≥0.60).

^b^ According to the Asian-Pacific guidelines, BMI is divided into four groups: underweight (<18.5 kg/m^2^), normal (18.5–22.9 kg/m^2^),

overweight (23.0–24.9 kg/m^2^), and obese (25–34.9 kg/m^2^).

**Table S2**. National trends in the prevalence of type 2 diabetes mellitus, stratified by central adiposity groups and β-coefficients of odds ratios comparing before and during the COVID–19 pandemic (weighted % [95% CI]).

| Variables | | Total |  | | | During the pandemic | | | Trends before the pandemic, β (95% CI) | Trends in the pandemic, β (95% CI) | β_diff_ between 2005–2019 and 2019–2022 (95% CI) |
| --- | --- | --- | --- | --- | --- | --- | --- | --- | --- | --- | --- |
|  |  |  | 2017 | 2018 | 2019 | 2020 | 2021 | 2022 |  |  |  |
| Overall | Healthy central adiposity | 5.87 (5.40 to 6.35) | 5.93 (4.86 to 7.00) | 5.52 (4.45 to 6.60) | 5.08 (4.01 to 6.16) | 5.77 (4.46 to 7.09) | 7.16 (5.78 to 8.54) | 5.80 (4.67 to 6.93) | 0.26 (-0.12 to 0.65) | -1.36 (-3.15 to 0.42) | -1.63 (-3.45 to 0.20) |
|  | Increased central adiposity | 17.04 (16.30 to 17.77) | 14.73 (12.90 to 16.55) | 16.21 (14.55 to 17.87) | 16.92 (15.33 to 18.51) | 18.05 (16.22 to 19.89) | 18.85 (16.72 to 20.98) | 17.07 (15.36 to 18.78) | **1.01 (0.39 to 1.62)** | -1.78 (-4.50 to 0.94) | -2.79 (-5.58 to 0.00) |
|  | High central adiposity | 31.31 (29.19 to 33.43) | 32.16 (26.00 to 38.32) | 27.20 (22.56 to 31.83) | 34.38 (29.37 to 39.38) | 37.16 (31.97 to 42.34) | 30.13 (25.05 to 35.21) | 26.70 (21.96 to 31.44) | 0.49 (-1.26 to 2.24) | -3.43 (-10.35 to 3.49) | -3.92 (-11.06 to 3.22) |
| **Healthy central adiposity** ^a^ | |  |  |  |  |  |  |  |  |  |  |
| Sex | Male | 8.49 (7.62 to 9.36) | 9.27 (7.32 to 11.22) | 8.50 (6.41 to 10.60) | 6.88 (5.07 to 8.68) | 8.29 (5.92 to 10.65) | 9.52 (6.92 to 12.12) | 8.31 (6.22 to 10.40) | 0.01 (-0.70 to 0.71) | -1.21 (-4.53 to 2.12) | -1.21 (-4.61 to 2.19) |
|  | Female | 3.78 (3.26 to 4.31) | 3.00 (1.79 to 4.21) | 3.12 (2.06 to 4.17) | 3.54 (2.38 to 4.71) | 4.03 (2.47 to 5.59) | 5.22 (3.77 to 6.67) | 3.97 (2.64 to 5.29) | **0.53 (0.11 to 0.95)** | -1.25 (-3.21 to 0.70) | -1.78 (-3.78 to 0.22) |
| Age (years) | 30–39 | 0.40 (0.09 to 0.70) | N/A | 0.46 (0.00 to 1.16) | 0.31 (0.00 to 0.78) | 0.38 (0.00 to 1.13) | 0.69 (0.00 to 1.66) | 0.64 (0.00 to 1.90) | 0.13 (-0.08 to 0.34) | -0.04 (-1.63 to 1.54) | -0.17 (-1.77 to 1.43) |
|  | 40–49 | 2.89 (2.17 to 3.61) | 3.65 (2.06 to 5.24) | 3.73 (1.44 to 6.03) | 2.52 (1.07 to 3.98) | 1.81 (0.16 to 3.45) | 4.67 (2.56 to 6.79) | 0.57 (0.00 to 1.49) | 0.00 (-0.59 to 0.59) | **-4.10 (-6.40 to -1.80)** | **-4.10 (-6.48 to -1.73)** |
|  | 50–59 | 9.26 (7.93 to 10.59) | 10.63 (7.30 to 13.97) | 8.14 (5.08 to 11.21) | 7.21 (4.63 to 9.79) | 11.88 (8.06 to 15.69) | 8.06 (4.92 to 11.20) | 9.70 (6.05 to 13.35) | -0.18 (-1.21 to 0.85) | 1.64 (-3.15 to 6.44) | 1.83 (-3.08 to 6.73) |
|  | ≥60 | 14.59 (13.09 to 16.08) | 13.08 (9.87 to 16.29) | 13.09 (10.20 to 15.99) | 16.48 (12.28 to 20.68) | 13.32 (9.22 to 17.43) | 17.12 (12.98 to 21.26) | 14.43 (10.83 to 18.03) | 0.90 (-0.26 to 2.07) | -2.69 (-8.16 to 2.78) | -3.59 (-9.18 to 2.00) |
| Region of residence | Urban | 5.46 (4.96 to 5.97) | 5.20 (4.18 to 6.22) | 5.66 (4.46 to 6.85) | 4.49 (3.38 to 5.61) | 5.68 (4.17 to 7.18) | 6.75 (5.25 to 8.25) | 5.03 (3.89 to 6.18) | 0.30 (-0.11 to 0.71) | -1.72 (-3.59 to 0.16) | **-2.02 (-3.94 to -0.09)** |
|  | Rural | 8.48 (7.02 to 9.94) | 10.82 (6.20 to 15.45) | 4.58 (2.34 to 6.82) | 8.57 (5.51 to 11.64) | 6.38 (3.80 to 8.97) | 9.57 (6.51 to 12.63) | 10.71 (6.24 to 15.17) | -0.06 (-1.25 to 1.13) | 1.14 (-4.25 to 6.53) | 1.20 (-4.33 to 6.72) |
| BMI group ^b^ | Underweight or normal weight | 6.47 (5.90 to 7.04) | 6.32 (4.96 to 7.68) | 6.16 (4.87 to 7.45) | 5.59 (4.32 to 6.86) | 5.82 (4.32 to 7.31) | 8.14 (6.47 to 9.81) | 6.76 (5.38 to 8.14) | 0.33 (-0.14 to 0.80) | -1.37 (-3.53 to 0.79) | -1.71 (-3.92 to 0.50) |
|  | Overweight | 4.02 (3.13 to 4.92) | 4.10 (2.25 to 5.94) | 4.71 (2.34 to 7.08) | 3.52 (1.38 to 5.66) | 5.61 (2.57 to 8.65) | 3.95 (1.87 to 6.02) | 2.11 (0.69 to 3.53) | 0.05 (-0.62 to 0.72) | -1.83 (-4.34 to 0.67) | -1.89 (-4.48 to 0.70) |
|  | Obese | 4.56 (2.47 to 6.65) | 7.96 (2.90 to 13.02) | 1.09 (0.00 to 3.26) | 2.78 (0.00 to 6.49) | 5.80 (0.00 to 12.63) | 4.37 (0.00 to 9.20) | 5.16 (0.00 to 13.18) | -0.41 (-1.98 to 1.17) | 0.79 (-8.56 to 10.15) | 1.20 (-8.29 to 10.69) |
| Level of education | Middle school or lower education | 14.08 (12.21 to 15.96) | 12.26 (8.64 to 15.89) | 13.03 (9.47 to 16.59) | 15.77 (10.35 to 21.19) | 12.39 (6.44 to 18.34) | 15.91 (11.08 to 20.74) | 16.98 (11.17 to 22.79) | 0.72 (-0.66 to 2.10) | 1.07 (-6.45 to 8.60) | 0.35 (-7.30 to 8.00) |
|  | College or higher education | 4.76 (4.28 to 5.23) | 4.79 (3.70 to 5.87) | 4.23 (3.13 to 5.34) | 3.89 (2.87 to 4.92) | 5.00 (3.70 to 6.29) | 6.07 (4.61 to 7.53) | 4.61 (3.53 to 5.69) | 0.33 (-0.07 to 0.72) | -1.46 (-3.27 to 0.35) | -1.79 (-3.64 to 0.06) |
| Household income | Lowest and second quartile | 7.86 (6.93 to 8.79) | 8.25 (5.81 to 10.69) | 7.87 (5.85 to 9.88) | 7.78 (5.64 to 9.92) | 5.12 (3.04 to 7.19) | 10.42 (7.64 to 13.20) | 7.41 (5.20 to 9.63) | 0.16 (-0.64 to 0.95) | -3.00 (-6.54 to 0.54) | -3.16 (-6.79 to 0.47) |
|  | Third and highest quartile | 4.97 (4.41 to 5.54) | 4.87 (3.67 to 6.07) | 4.31 (3.13 to 5.48) | 3.68 (2.50 to 4.85) | 6.04 (4.32 to 7.75) | 5.87 (4.19 to 7.56) | 5.11 (3.77 to 6.45) | 0.36 (-0.10 to 0.81) | -0.77 (-2.91 to 1.38) | -1.12 (-3.32 to 1.07) |
| Smoking status | Smoker or ex-smoker | 8.04 (7.16 to 8.93) | 8.43 (6.48 to 10.38) | 7.65 (5.46 to 9.84) | 7.15 (5.27 to 9.04) | 7.29 (5.09 to 9.49) | 9.71 (7.05 to 12.37) | 7.96 (5.82 to 10.10) | 0.20 (-0.51 to 0.92) | -1.75 (-5.15 to 1.66) | -1.95 (-5.43 to 1.53) |
|  | Non-smoker | 4.32 (3.77 to 4.87) | 4.01 (2.66 to 5.37) | 3.96 (2.82 to 5.10) | 3.55 (2.38 to 4.73) | 4.76 (3.05 to 6.47) | 5.40 (3.98 to 6.81) | 4.31 (2.93 to 5.69) | 0.35 (-0.09 to 0.79) | -1.09 (-3.06 to 0.88) | -1.44 (-3.46 to 0.58) |
| **Increased central adiposity** ^a^ | |  |  |  |  |  |  |  |  |  |  |
| Sex | Male | 18.40 (17.32 to 19.48) | 15.48 (12.87 to 18.09) | 16.96 (14.41 to 19.51) | 18.63 (16.24 to 21.02) | 19.37 (16.57 to 22.17) | 21.54 (18.59 to 24.48) | 17.82 (15.37 to 20.27) | **1.45 (0.58 to 2.32)** | -3.71 (-7.54 to 0.11) | **-5.16 (-9.08 to -1.24)** |
|  | Female | 15.27 (14.33 to 16.20) | 13.78 (11.58 to 15.99) | 15.27 (13.11 to 17.42) | 14.83 (12.89 to 16.78) | 16.26 (13.79 to 18.74) | 15.31 (12.75 to 17.88) | 16.01 (13.59 to 18.44) | 0.40 (-0.36 to 1.16) | 0.70 (-2.82 to 4.23) | 0.30 (-3.30 to 3.91) |
| Age (years) | 30–39 | 4.69 (3.53 to 5.85) | 4.71 (1.79 to 7.62) | 4.33 (1.51 to 7.14) | 5.63 (2.79 to 8.47) | 6.11 (3.05 to 9.16) | 3.42 (0.71 to 6.12) | 3.70 (1.11 to 6.29) | -0.09 (-1.00 to 0.81) | 0.29 (-3.44 to 4.02) | 0.38 (-3.45 to 4.22) |
|  | 40–49 | 10.49 (9.02 to 11.97) | 6.99 (4.15 to 9.84) | 11.00 (7.51 to 14.49) | 12.31 (8.25 to 16.37) | 11.35 (7.78 to 14.93) | 12.83 (8.92 to 16.74) | 7.94 (4.65 to 11.22) | **1.17 (0.08 to 2.26)** | -4.90 (-9.98 to 0.19) | **-6.07 (-11.27 to -0.86)** |
|  | 50–59 | 17.47 (15.90 to 19.04) | 15.81 (12.03 to 19.58) | 16.67 (12.82 to 20.51) | 14.67 (11.48 to 17.85) | 19.32 (15.41 to 23.23) | 18.50 (14.38 to 22.61) | 19.67 (15.50 to 23.83) | 0.82 (-0.42 to 2.06) | 1.17 (-4.66 to 7.00) | 0.35 (-5.61 to 6.31) |
|  | ≥60 | 24.97 (23.83 to 26.12) | 21.79 (18.60 to 24.98) | 23.38 (20.79 to 25.98) | 25.11 (22.86 to 27.37) | 26.21 (23.30 to 29.12) | 28.19 (25.17 to 31.20) | 24.53 (21.69 to 27.37) | **1.56 (0.61 to 2.51)** | -3.66 (-7.78 to 0.47) | **-5.22 (-9.46 to -0.99)** |
| Region of residence | Urban | 16.47 (15.65 to 17.30) | 14.49 (12.46 to 16.53) | 15.35 (13.55 to 17.16) | 16.00 (14.26 to 17.73) | 17.84 (15.76 to 19.92) | 17.78 (15.36 to 20.20) | 16.99 (14.99 to 19.00) | **0.91 (0.22 to 1.60)** | -0.78 (-3.91 to 2.35) | -1.69 (-4.90 to 1.51) |
|  | Rural | 19.63 (17.95 to 21.31) | 15.79 (11.49 to 20.08) | 20.38 (15.88 to 24.88) | 20.78 (16.91 to 24.65) | 19.15 (15.48 to 22.81) | 23.61 (19.18 to 28.05) | 17.42 (14.22 to 20.63) | **1.44 (0.06 to 2.83)** | **-6.19 (-11.64 to -0.74)** | **-7.63 (-13.26 to -2.01)** |
| BMI group ^b^ | Underweight or normal weight | 20.77 (18.82 to 22.72) | 20.71 (15.46 to 25.96) | 20.24 (15.51 to 24.97) | 15.78 (12.26 to 19.30) | 23.76 (18.88 to 28.63) | 20.99 (15.75 to 26.23) | 24.26 (19.20 to 29.32) | 0.53 (-1.09 to 2.15) | 3.27 (-3.98 to 10.53) | 2.75 (-4.69 to 10.18) |
|  | Overweight | 16.78 (15.52 to 18.04) | 13.12 (9.97 to 16.28) | 18.37 (15.10 to 21.64) | 16.56 (13.96 to 19.16) | 18.24 (14.58 to 21.90) | 17.24 (14.06 to 20.42) | 16.70 (13.79 to 19.61) | 0.79 (-0.22 to 1.80) | -0.54 (-4.83 to 3.75) | -1.33 (-5.74 to 3.08) |
|  | Obese | 16.27 (15.30 to 17.24) | 14.36 (12.26 to 16.46) | 14.36 (12.42 to 16.30) | 17.53 (15.08 to 19.97) | 16.56 (14.11 to 19.01) | 19.19 (16.34 to 22.04) | 15.51 (13.18 to 17.84) | **1.19 (0.41 to 1.97)** | **-3.68 (-7.34 to -0.02)** | **-4.87 (-8.61 to -1.12)** |
| Level of education | Middle school or lower education | 24.31 (23.00 to 25.62) | 19.76 (16.76 to 22.76) | 22.81 (19.73 to 25.89) | 25.46 (22.77 to 28.14) | 27.18 (23.55 to 30.81) | 27.29 (23.60 to 30.98) | 24.44 (20.99 to 27.89) | **1.97 (0.92 to 3.03)** | -2.85 (-7.88 to 2.18) | -4.82 (-9.96 to 0.32) |
|  | College or higher education | 14.34 (13.50 to 15.18) | 12.05 (10.09 to 14.01) | 13.37 (11.41 to 15.34) | 13.67 (11.82 to 15.52) | 15.25 (13.20 to 17.29) | 16.08 (13.77 to 18.39) | 14.85 (12.82 to 16.88) | **1.00 (0.32 to 1.67)** | -1.23 (-4.29 to 1.83) | -2.23 (-5.36 to 0.90) |
| Household income | Lowest and second quartile | 21.11 (19.98 to 22.25) | 18.52 (15.72 to 21.32) | 20.10 (17.54 to 22.67) | 19.83 (17.35 to 22.32) | 23.25 (20.46 to 26.04) | 24.57 (21.37 to 27.77) | 20.41 (17.51 to 23.31) | **1.53 (0.61 to 2.44)** | -4.16 (-8.46 to 0.14) | **-5.69 (-10.08 to -1.29)** |
|  | Third and highest quartile | 14.13 (13.24 to 15.01) | 11.65 (9.59 to 13.71) | 13.25 (11.16 to 15.35) | 14.70 (12.70 to 16.69) | 14.77 (12.54 to 17.00) | 14.97 (12.69 to 17.25) | 14.81 (12.62 to 16.99) | **0.79 (0.10 to 1.49)** | -0.17 (-3.31 to 2.98) | -0.96 (-4.18 to 2.26) |
| Smoking status | Smoker or ex-smoker | 18.65 (17.50 to 19.81) | 15.56 (12.83 to 18.29) | 16.56 (13.94 to 19.17) | 18.39 (15.75 to 21.02) | 19.84 (16.91 to 22.78) | 22.25 (19.09 to 25.41) | 18.60 (15.93 to 21.27) | **1.68 (0.75 to 2.60)** | -3.65 (-7.78 to 0.47) | **-5.33 (-9.55 to -1.10)** |
|  | Non-smoker | 15.48 (14.57 to 16.40) | 13.97 (11.69 to 16.26) | 15.87 (13.80 to 17.94) | 15.53 (13.49 to 17.56) | 16.30 (14.12 to 18.49) | 15.53 (12.92 to 18.14) | 15.54 (13.28 to 17.81) | 0.35 (-0.41 to 1.11) | 0.01 (-3.43 to 3.46) | -0.33 (-3.86 to 3.19) |
| **High central adiposity** ^a^ | |  |  |  |  |  |  |  |  |  |  |
| Sex | Male | 35.78 (31.78 to 39.77) | 37.27 (24.71 to 49.83) | 33.10 (22.83 to 43.38) | 44.19 (33.84 to 54.54) | 40.64 (31.18 to 50.09) | 33.04 (22.95 to 43.13) | 29.09 (20.82 to 37.35) | -0.38 (-3.78 to 3.03) | -3.95 (-16.95 to 9.04) | -3.58 (-17.01 to 9.86) |
|  | Female | 28.97 (26.47 to 31.47) | 30.41 (23.43 to 37.39) | 24.28 (18.57 to 29.99) | 30.18 (24.55 to 35.82) | 34.88 (29.21 to 40.55) | 28.62 (22.88 to 34.35) | 25.09 (18.72 to 31.46) | 0.61 (-1.38 to 2.60) | -3.53 (-12.06 to 5.01) | -4.14 (-12.90 to 4.62) |
| Age (years) | 30–39 | 19.07 (11.17 to 26.97) | 9.01 (0.00 to 21.67) | 18.22 (1.57 to 34.87) | 10.23 (0.00 to 24.45) | 24.91 (8.87 to 40.96) | 25.74 (1.58 to 49.91) | 20.26 (0.58 to 39.94) | 3.87 (-2.76 to 10.50) | -5.49 (-36.53 to 25.56) | -9.36 (-41.10 to 22.39) |
|  | 40–49 | 30.23 (23.51 to 36.94) | 34.50 (17.09 to 51.90) | 33.58 (10.66 to 56.50) | 24.56 (9.51 to 39.61) | 37.03 (20.67 to 53.39) | 29.61 (13.70 to 45.52) | 24.58 (10.11 to 39.04) | -0.45 (-5.87 to 4.96) | -5.03 (-26.46 to 16.40) | -4.58 (-26.68 to 17.52) |
|  | 50–59 | 34.47 (28.87 to 40.06) | 29.79 (14.99 to 44.60) | 10.14 (0.81 to 19.47) | 42.63 (29.04 to 56.23) | 38.46 (26.28 to 50.63) | 35.91 (21.20 to 50.61) | 34.08 (21.54 to 46.63) | 3.28 (-1.27 to 7.83) | -1.83 (-21.08 to 17.43) | -5.11 (-24.89 to 14.68) |
|  | ≥60 | 32.46 (30.05 to 34.87) | 35.31 (28.19 to 42.43) | 31.00 (25.60 to 36.39) | 36.43 (31.31 to 41.56) | 38.51 (32.14 to 44.89) | 29.36 (24.85 to 33.88) | 25.78 (19.28 to 32.28) | -0.57 (-2.41 to 1.26) | -3.58 (-11.46 to 4.30) | -3.01 (-11.10 to 5.08) |
| Region of residence | Urban | 31.07 (28.60 to 33.55) | 31.58 (24.85 to 38.31) | 24.70 (19.57 to 29.83) | 35.54 (29.59 to 41.49) | 37.64 (31.83 to 43.46) | 30.51 (24.35 to 36.68) | 25.95 (20.29 to 31.61) | 1.04 (-0.98 to 3.06) | -4.56 (-12.90 to 3.78) | -5.60 (-14.18 to 2.98) |
|  | Rural | 32.16 (28.06 to 36.26) | 34.05 (20.13 to 47.97) | 39.63 (28.48 to 50.78) | 30.36 (22.13 to 38.60) | 35.41 (24.46 to 46.36) | 28.92 (20.59 to 37.24) | 29.25 (20.20 to 38.31) | -1.41 (-4.86 to 2.05) | 0.34 (-11.91 to 12.59) | 1.74 (-10.99 to 14.47) |
| BMI group ^b^ | Underweight or normal weight | 31.35 (6.06 to 56.63) | 24.22 (0.00 to 66.06) | 21.80 (0.00 to 63.60) | 21.05 (0.00 to 61.77) | N/A | 50.62 (0.00 to 100.00) | N/A | 7.10 (-9.33 to 23.52) | N/A | N/A |
|  | Overweight | 25.09 (15.89 to 34.30) | 12.17 (0.00 to 27.68) | 31.12 (0.00 to 63.07) | 31.30 (8.97 to 53.62) | 50.94 (22.51 to 79.38) | 18.80 (5.16 to 32.45) | 8.53 (0.00 to 24.06) | 2.81 (-3.33 to 8.96) | -10.27 (-30.86 to 10.32) | -13.09 (-34.57 to 8.40) |
|  | Obese | 31.55 (29.36 to 33.73) | 33.28 (26.63 to 39.92) | 27.13 (22.23 to 32.04) | 34.60 (29.47 to 39.72) | 36.69 (31.48 to 41.91) | 30.43 (25.11 to 35.74) | 27.26 (22.43 to 32.09) | 0.33 (-1.51 to 2.17) | -3.16 (-10.31 to 3.99) | -3.49 (-10.88 to 3.89) |
| Level of education | Middle school or lower education | 31.90 (29.19 to 34.60) | 35.50 (27.65 to 43.35) | 28.53 (22.33 to 34.73) | 35.59 (29.75 to 41.42) | 38.31 (31.12 to 45.50) | 30.17 (24.51 to 35.82) | 23.07 (16.12 to 30.02) | -0.18 (-2.31 to 1.95) | -7.10 (-16.01 to 1.82) | -6.92 (-16.08 to 2.25) |
|  | College or higher education | 30.71 (27.31 to 34.10) | 26.90 (17.94 to 35.87) | 25.49 (17.24 to 33.74) | 32.92 (24.10 to 41.74) | 36.14 (27.94 to 44.34) | 30.09 (21.60 to 38.59) | 29.40 (22.66 to 36.14) | 1.49 (-1.30 to 4.28) | -0.70 (-11.50 to 10.11) | -2.19 (-13.35 to 8.97) |
| Household income | Lowest and second quartile | 32.71 (30.22 to 35.21) | 36.96 (29.89 to 44.03) | 31.49 (25.61 to 37.38) | 31.44 (25.84 to 37.04) | 36.31 (29.88 to 42.73) | 31.86 (26.09 to 37.64) | 29.28 (23.37 to 35.20) | -0.50 (-2.51 to 1.51) | -2.58 (-10.81 to 5.65) | -2.08 (-10.56 to 6.39) |
|  | Third and highest quartile | 29.30 (25.68 to 32.92) | 22.71 (12.99 to 32.43) | 19.68 (11.71 to 27.66) | 39.03 (29.17 to 48.89) | 38.21 (29.45 to 46.96) | 27.81 (19.76 to 35.85) | 23.65 (16.06 to 31.24) | 2.26 (-0.57 to 5.08) | -4.16 (-15.18 to 6.86) | -6.41 (-17.79 to 4.96) |
| Smoking status | Smoker or ex-smoker | 35.46 (31.54 to 39.37) | 35.40 (24.60 to 46.20) | 27.93 (18.48 to 37.38) | 47.84 (38.08 to 57.60) | 41.62 (31.68 to 51.55) | 33.77 (23.94 to 43.60) | 28.78 (20.71 to 36.84) | 0.73 (-2.46 to 3.93) | -4.99 (-17.66 to 7.68) | -5.73 (-18.79 to 7.34) |
|  | Non-smoker | 29.17 (26.66 to 31.69) | 30.77 (23.87 to 37.68) | 26.81 (20.84 to 32.78) | 29.11 (23.31 to 34.90) | 34.78 (28.76 to 40.79) | 28.20 (22.56 to 33.84) | 25.31 (19.01 to 31.61) | 0.25 (-1.72 to 2.23) | -2.89 (-11.31 to 5.54) | -3.14 (-11.80 to 5.51) |

Abbreviations: BMI, body mass index; CI, confidence interval; KNHANES, Korea National Health and Nutrition Examination Survey.

Beta values were multiplied by 100 due to their small magnitudes.
The values in bold font represent significant variance (p<0.05).

^a^ According to the guidelines from the National Institute for Health and Care Excellence, central adiposity is divided into three groups: healthy central adiposity (waist-to-height ratio 0.40–0.49), increased central adiposity (waist-to-height ratio 0.50–0.59), and high central adiposity (waist-to-height ratio ≥0.60).

^b^ According to the Asian-Pacific guidelines, BMI is divided into four groups: underweight (<18.5 kg/m^2^), normal (18.5–22.9 kg/m^2^),

overweight (23.0–24.9 kg/m^2^), and obese (25.0–34.9 kg/m^2^).

**Table S3**. Weighted odds ratios in the periods for prevalence of type 2 diabetes mellitus, stratified by central adiposity groups (weighted % [95% CI]).

| Variables | | 2005–2007 (reference) versus 2008–2010 | | 2008–2010 (reference) versus 2011–2013 | | 2011–2013 (reference) versus 2014–2016 | | 2014–2016 (reference) versus 2017–2019 | | 2017–2019 (reference) versus 2020 | | 2020 (reference) versus 2021 | | 2021 (reference) versus 2022 | |
| --- | --- | --- | --- | --- | --- | --- | --- | --- | --- | --- | --- | --- | --- | --- | --- |
|  |  | Weighted OR (95% CI) | P-value | Weighted OR (95% CI) | P-value | Weighted OR (95% CI) | P-value | Weighted OR (95% CI) | P-value | Weighted OR (95% CI) | P-value | Weighted OR (95% CI) | P-value | Weighted OR (95% CI) | P-value |
| Overall | Healthy central adiposity | **1.38 (1.07 to 1.79)** | **0.014** | **1.25 (1.03 to 1.51)** | **0.024** | 0.91 (0.75 to 1.10) | 0.317 | 1.09 (0.91 to 1.31) | 0.345 | 1.05 (0.80 to 1.37) | 0.742 | 1.26 (0.91 to 1.74) | 0.160 | 0.80 (0.60 to 1.07) | 0.131 |
|  | Increased central adiposity | 1.12 (0.95 to 1.31) | 0.172 | 1.04 (0.93 to 1.17) | 0.481 | 1.12 (0.99 to 1.26) | 0.063 | **1.16 (1.04 to 1.30)** | **0.008** | **1.16 (1.01 to 1.34)** | **0.052** | 1.05 (0.87 to 1.27) | 0.582 | 0.89 (0.74 to 1.07) | 0.196 |
|  | High central adiposity | 1.34 (0.98 to 1.85) | 0.070 | 1.07 (0.83 to 1.38) | 0.586 | 0.94 (0.73 to 1.21) | 0.634 | **1.55 (1.24 to 1.93)** | **<.001** | 1.29 (0.99 to 1.67) | 0.058 | 0.73 (0.52 to 1.02) | 0.063 | 0.85 (0.60 to 1.19) | 0.331 |
| **Healthy central adiposity** ^a^ | |  |  |  |  |  |  |  |  |  |  |  |  |  |  |
| Sex | Male | 1.21 (0.87 to 1.66) | 0.254 | **1.38 (1.09 to 1.74)** | **0.008** | 0.87 (0.69 to 1.10) | 0.240 | 1.15 (0.92 to 1.44) | 0.228 | 1.00 (0.71 to 1.41) | 0.997 | 1.16 (0.76 to 1.78) | 0.483 | 0.86 (0.57 to 1.29) | 0.472 |
|  | Female | **1.69 (1.10 to 2.58)** | **0.016** | 1.05 (0.77 to 1.41) | 0.770 | 1.03 (0.75 to 1.40) | 0.877 | 1.04 (0.77 to 1.41) | 0.801 | 1.27 (0.81 to 1.99) | 0.304 | 1.31 (0.80 to 2.16) | 0.285 | 0.75 (0.48 to 1.18) | 0.213 |
| Age, years | 30–39 | 1.87 (0.73 to 4.84) | 0.195 | 0.99 (0.53 to 1.85) | 0.970 | 0.62 (0.27 to 1.38) | 0.239 | 0.30 (0.08 to 1.04) | 0.058 | 1.51 (0.16 to 14.07) | 0.719 | 1.80 (0.16 to 20.06) | 0.632 | 0.94 (0.08 to 10.62) | 0.957 |
|  | 40–49 | 1.54 (0.94 to 2.53) | 0.090 | 0.82 (0.55 to 1.22) | 0.324 | 0.98 (0.62 to 1.55) | 0.928 | 0.92 (0.57 to 1.48) | 0.724 | 0.53 (0.20 to 1.42) | 0.210 | 2.67 (0.95 to 7.50) | 0.063 | **0.12 (0.02 to 0.63)** | **0.012** |
|  | 50–59 | 0.99 (0.56 to 1.74) | 0.962 | **1.47 (1.02 to 2.12)** | **0.039** | 0.69 (0.48 to 1.00) | 0.051 | **1.44 (1.01 to 2.05)** | **0.044** | 1.41 (0.92 to 2.16) | 0.120 | 0.65 (0.37 to 1.14) | 0.131 | 1.23 (0.68 to 2.22) | 0.499 |
|  | ≥60 | 1.16 (0.81 to 1.65) | 0.422 | 1.30 (1.00 to 1.70) | 0.053 | 1.06 (0.82 to 1.38) | 0.643 | 0.91 (0.72 to 1.16) | 0.454 | 0.94 (0.64 to 1.39) | 0.770 | 1.34 (0.85 to 2.12) | 0.203 | 0.82 (0.54 to 1.23) | 0.332 |
| Region of residence | Urban | **1.38 (1.02 to 1.86)** | **0.038** | 1.20 (0.97 to 1.49) | 0.102 | 1.00 (0.81 to 1.25) | 0.983 | 1.01 (0.83 to 1.23) | 0.949 | 1.11 (0.81 to 1.51) | 0.514 | 1.20 (0.83 to 1.74) | 0.326 | 0.73 (0.52 to 1.03) | 0.069 |
|  | Rural | 1.47 (0.89 to 2.43) | 0.132 | 1.45 (0.98 to 2.14) | 0.066 | **0.60 (0.39 to 0.93)** | **0.022** | **1.67 (1.08 to 2.60)** | **0.022** | 0.78 (0.47 to 1.32) | 0.354 | 1.55 (0.89 to 2.70) | 0.119 | **1.13 (0.63 to 2.03)** | **0.674** |
| BMI group ^b^ | Underweight or normal weight | 1.29 (0.96 to 1.72) | 0.092 | **1.28 (1.02 to 1.61)** | **0.037** | 0.93 (0.74 to 1.17) | 0.517 | 1.16 (0.95 to 1.43) | 0.146 | 0.96 (0.71 to 1.31) | 0.803 | **1.43 (1.01 to 2.05)** | **0.046** | 0.82 (0.60 to 1.12) | 0.208 |
|  | Overweight | 1.81 (0.99 to 3.32) | 0.055 | 1.25 (0.86 to 1.80) | 0.241 | 0.67 (0.45 to 1.00) | 0.052 | 1.01 (0.65 to 1.57) | 0.952 | 1.37 (0.72 to 2.61) | 0.344 | 0.69 (0.31 to 1.53) | 0.360 | 0.53 (0.22 to 1.26) | 0.148 |
|  | Obese | 1.28 (0.41 to 4.05) | 0.674 | 0.90 (0.38 to 2.13) | 0.815 | 1.99 (0.85 to 4.63) | 0.111 | 0.55 (0.23 to 1.31) | 0.179 | 1.40 (0.35 to 5.56) | 0.635 | 0.74 (0.14 to 4.06) | 0.730 | 1.19 (0.16 to 8.84) | 0.864 |
| Level of education | Middle school or lower education | **2.78 (2.09 to 3.70)** | **<.001** | 1.26 (0.95 to 1.67) | 0.102 | 0.92 (0.67 to 1.25) | 0.589 | **1.40 (1.04 to 1.88)** | **0.027** | 0.92 (0.52 to 1.63) | 0.771 | 1.34 (0.70 to 2.57) | 0.379 | 1.08 (0.63 to 1.87) | 0.779 |
|  | College or higher education | 0.99 (0.59 to 1.65) | 0.959 | **1.30 (1.01 to 1.68)** | **0.042** | 0.97 (0.76 to 1.23) | 0.773 | 1.04 (0.83 to 1.30) | 0.719 | 1.17 (0.85 to 1.59) | 0.333 | 1.23 (0.85 to 1.78) | 0.277 | 0.75 (0.53 to 1.07) | 0.107 |
| Household income | Lowest and second quartile | **1.61 (1.14 to 2.28)** | **0.007** | 1.14 (0.89 to 1.46) | 0.305 | 1.06 (0.82 to 1.38) | 0.639 | 1.04 (0.81 to 1.35) | 0.739 | **0.62 (0.39 to 0.99)** | **0.046** | **2.16 (1.27 to 3.66)** | **0.005** | 0.69 (0.45 to 1.07) | 0.094 |
|  | Third and highest quartile | 1.23 (0.84 to 1.79) | 0.283 | **1.33 (1.02 to 1.73)** | **0.033** | 0.85 (0.65 to 1.11) | 0.229 | 1.12 (0.87 to 1.43) | 0.383 | **1.42 (1.01 to 2.01)** | **0.045** | 0.97 (0.64 to 1.49) | 0.892 | 0.86 (0.57 to 1.30) | 0.479 |
| Smoking status | Smoker or ex-smoker | 1.17 (0.85 to 1.62) | 0.335 | **1.39 (1.07 to 1.79)** | **0.012** | 0.94 (0.73 to 1.20) | 0.606 | 1.05 (0.83 to 1.34) | 0.663 | 0.93 (0.65 to 1.34) | 0.705 | 1.37 (0.88 to 2.13) | 0.164 | 0.81 (0.53 to 1.23) | 0.310 |
|  | Non-smoker | **1.71 (1.10 to 2.64)** | **0.016** | 1.09 (0.82 to 1.45) | 0.536 | 0.90 (0.67 to 1.20) | 0.482 | 1.17 (0.88 to 1.55) | 0.283 | 1.25 (0.82 to 1.89) | 0.302 | 1.14 (0.72 to 1.82) | 0.572 | 0.79 (0.51 to 1.22) | 0.283 |
| **Increased central adiposity** ^a^ | |  |  |  |  |  |  |  |  |  |  |  |  |  |  |
| Sex | Male | 1.00 (0.81 to 1.22) | 0.969 | 1.05 (0.90 to 1.23) | 0.539 | 1.11 (0.94 to 1.30) | 0.219 | 1.13 (0.97 to 1.31) | 0.128 | 1.17 (0.95 to 1.43) | 0.145 | 1.14 (0.89 to 1.46) | 0.292 | 0.79 (0.62 to 1.01) | 0.055 |
|  | Female | **1.30 (1.05 to 1.60)** | **0.015** | 1.03 (0.87 to 1.22) | 0.735 | 1.13 (0.96 to 1.33) | 0.146 | **1.19 (1.03 to 1.38)** | **0.018** | 1.13 (0.92 to 1.40) | 0.249 | 0.93 (0.71 to 1.23) | 0.612 | 1.06 (0.81 to 1.38) | 0.696 |
| Age, years | 30–39 | 0.66 (0.35 to 1.26) | 0.211 | 1.07 (0.63 to 1.81) | 0.814 | 1.20 (0.67 to 2.15) | 0.541 | 1.12 (0.65 to 1.93) | 0.683 | 1.26 (0.66 to 2.38) | 0.486 | 0.54 (0.20 to 1.46) | 0.224 | 1.09 (0.37 to 3.24) | 0.880 |
|  | 40–49 | 1.01 (0.73 to 1.40) | 0.934 | 1.03 (0.75 to 1.43) | 0.852 | 1.24 (0.89 to 1.73) | 0.206 | 1.02 (0.75 to 1.38) | 0.922 | 1.13 (0.74 to 1.72) | 0.575 | 1.15 (0.70 to 1.89) | 0.582 | 0.59 (0.33 to 1.03) | 0.064 |
|  | 50–59 | 1.00 (0.76 to 1.32) | 0.992 | 0.96 (0.77 to 1.19) | 0.691 | 1.04 (0.83 to 1.29) | 0.747 | 1.10 (0.89 to 1.37) | 0.387 | 1.29 (0.96 to 1.72) | 0.089 | 0.95 (0.66 to 1.37) | 0.773 | 1.08 (0.74 to 1.57) | 0.693 |
|  | ≥60 | **1.28 (1.05 to 1.56)** | **0.015** | 1.03 (0.89 to 1.19) | 0.692 | 1.08 (0.93 to 1.25) | 0.334 | **1.16 (1.02 to 1.32)** | **0.026** | 1.16 (0.97 to 1.38) | 0.107 | 1.11 (0.89 to 1.37) | 0.359 | 0.83 (0.67 to 1.02) | 0.082 |
| Region of residence | Urban | 1.10 (0.91 to 1.32) | 0.324 | 0.99 (0.87 to 1.13) | 0.860 | 1.11 (0.97 to 1.26) | 0.131 | **1.14 (1.01 to 1.29)** | **0.039** | **1.20 (1.02 to 1.42)** | **0.032** | 1.00 (0.80 to 1.24) | 0.970 | 0.95 (0.76 to 1.18) | 0.622 |
|  | Rural | 1.18 (0.90 to 1.54) | 0.230 | 1.24 (0.98 to 1.57) | 0.074 | 1.19 (0.92 to 1.55) | 0.181 | 1.25 (0.98 to 1.60) | 0.076 | 1.00 (0.75 to 1.34) | 0.993 | 1.31 (0.93 to 1.84) | 0.126 | **0.68 (0.49 to 0.95)** | **0.024** |
| BMI group ^b^ | Underweight or normal weight | **1.55 (1.06 to 2.27)** | **0.025** | 1.24 (0.95 to 1.62) | 0.120 | 0.90 (0.68 to 1.17) | 0.419 | 1.22 (0.95 to 1.57) | 0.114 | **1.39 (1.01 to 1.91)** | **0.047** | 0.85 (0.56 to 1.29) | 0.447 | 1.21 (0.79 to 1.83) | 0.378 |
|  | Overweight | 0.87 (0.68 to 1.12) | 0.277 | 1.08 (0.88 to 1.32) | 0.487 | 1.19 (0.98 to 1.45) | 0.082 | 1.16 (0.97 to 1.39) | 0.113 | 1.16 (0.89 to 1.52) | 0.273 | 0.93 (0.67 to 1.30) | 0.683 | 0.96 (0.71 to 1.31) | 0.804 |
|  | Obese | 1.19 (0.96 to 1.48) | 0.122 | 0.98 (0.84 to 1.14) | 0.788 | 1.15 (0.98 to 1.35) | 0.087 | 1.14 (0.99 to 1.32) | 0.074 | 1.09 (0.89 to 1.34) | 0.402 | 1.20 (0.93 to 1.55) | 0.171 | **0.77 (0.60 to 1.00)** | **0.048** |
| Level of education | Middle school or lower education | **1.29 (1.09 to 1.53)** | **0.004** | 1.04 (0.89 to 1.22) | 0.592 | **1.22 (1.04 to 1.43)** | **0.017** | **1.27 (1.09 to 1.47)** | **0.002** | **1.28 (1.04 to 1.57)** | **0.019** | 1.01 (0.78 to 1.30) | 0.967 | 0.86 (0.66 to 1.12) | 0.266 |
|  | College or higher education | 1.25 (0.89 to 1.77) | 0.202 | 1.08 (0.91 to 1.29) | 0.392 | 1.11 (0.94 to 1.31) | 0.240 | 1.14 (0.98 to 1.32) | 0.085 | 1.19 (0.99 to 1.44) | 0.065 | 1.07 (0.84 to 1.35) | 0.594 | 0.91 (0.72 to 1.15) | 0.428 |
| Household income | Lowest and second quartile | **1.30 (1.07 to 1.59)** | **0.008** | 1.00 (0.86 to 1.16) | 0.952 | **1.25 (1.08 to 1.45)** | **0.004** | 1.13 (0.98 to 1.31) | 0.082 | **1.25 (1.04 to 1.51)** | **0.019** | 1.08 (0.85 to 1.36) | 0.546 | 0.79 (0.62 to 1.01) | 0.058 |
|  | Third and highest quartile | 0.95 (0.76 to 1.19) | 0.644 | 1.10 (0.93 to 1.30) | 0.280 | 1.01 (0.85 to 1.20) | 0.889 | **1.20 (1.02 to 1.40)** | **0.026** | 1.13 (0.92 to 1.39) | 0.240 | 1.02 (0.79 to 1.31) | 0.900 | 0.99 (0.77 to 1.27) | 0.916 |
| Smoking status | Smoker or ex-smoker | 1.00 (0.81 to 1.24) | 0.996 | 1.09 (0.92 to 1.28) | 0.320 | 1.09 (0.93 to 1.29) | 0.279 | 1.12 (0.96 to 1.31) | 0.157 | 1.22 (0.98 to 1.50) | 0.072 | 1.16 (0.90 to 1.49) | 0.264 | 0.80 (0.62 to 1.03) | 0.081 |
|  | Non-smoker | **1.25 (1.02 to 1.54)** | **0.033** | 1.00 (0.85 to 1.17) | 0.971 | 1.15 (0.98 to 1.36) | 0.084 | **1.20 (1.04 to 1.39)** | **0.013** | 1.09 (0.90 to 1.32) | 0.372 | 0.94 (0.73 to 1.23) | 0.666 | 1.00 (0.77 to 1.30) | 0.994 |
| **High central adiposity** ^a^ | |  |  |  |  |  |  |  |  |  |  |  |  |  |  |
| Sex | Male | 1.04 (0.52 to 2.10) | 0.906 | 1.05 (0.61 to 1.83) | 0.856 | 1.36 (0.81 to 2.28) | 0.247 | **1.59 (1.05 to 2.41)** | **0.028** | 1.09 (0.68 to 1.73) | 0.725 | 0.72 (0.40 to 1.30) | 0.276 | 0.83 (0.45 to 1.52) | 0.548 |
|  | Female | **1.42 (1.01 to 2.02)** | **0.047** | 1.07 (0.81 to 1.42) | 0.613 | 0.83 (0.62 to 1.11) | 0.201 | **1.49 (1.15 to 1.94)** | **0.003** | 1.34 (0.99 to 1.82) | 0.056 | 0.75 (0.51 to 1.09) | 0.129 | 0.84 (0.54 to 1.30) | 0.420 |
| Age, years | 30–39 | 3.43 (0.40 to 29.14) | 0.259 | 0.60 (0.13 to 2.78) | 0.514 | 2.80 (0.60 to 13.05) | 0.190 | 1.06 (0.35 to 3.21) | 0.912 | 2.15 (0.66 to 7.04) | 0.206 | 1.05 (0.24 to 4.60) | 0.954 | 0.73 (0.13 to 4.21) | 0.727 |
|  | 40–49 | 1.42 (0.37 to 5.55) | 0.611 | 1.39 (0.56 to 3.44) | 0.473 | 1.62 (0.71 to 3.69) | 0.251 | 1.37 (0.65 to 2.86) | 0.406 | 1.37 (0.60 to 3.11) | 0.454 | 0.72 (0.26 to 1.98) | 0.519 | 0.78 (0.26 to 2.30) | 0.645 |
|  | 50–59 | 1.36 (0.67 to 2.77) | 0.393 | 0.95 (0.50 to 1.79) | 0.863 | 0.69 (0.34 to 1.38) | 0.292 | **2.32 (1.24 to 4.36)** | **0.009** | 1.33 (0.70 to 2.53) | 0.377 | 0.90 (0.40 to 2.03) | 0.792 | 0.92 (0.40 to 2.15) | 0.852 |
|  | ≥60 | 1.34 (0.94 to 1.92) | 0.106 | 1.09 (0.83 to 1.44) | 0.538 | 0.86 (0.64 to 1.16) | 0.325 | **1.46 (1.13 to 1.89)** | **0.004** | 1.20 (0.88 to 1.63) | 0.250 | **0.66 (0.47 to 0.95)** | **0.023** | 0.84 (0.56 to 1.25) | 0.380 |
| Region of residence | Urban | **1.61 (1.09 to 2.39)** | **0.017** | 1.11 (0.82 to 1.49) | 0.497 | 0.84 (0.63 to 1.14) | 0.265 | **1.55 (1.20 to 2.01)** | **0.001** | **1.35 (1.00 to 1.82)** | **0.047** | 0.73 (0.49 to 1.08) | 0.110 | 0.80 (0.53 to 1.21) | 0.283 |
|  | Rural | 0.86 (0.51 to 1.46) | 0.580 | 0.96 (0.60 to 1.53) | 0.861 | 1.25 (0.78 to 2.01) | 0.359 | **1.57 (1.02 to 2.44)** | **0.043** | 1.07 (0.63 to 1.83) | 0.798 | 0.74 (0.40 to 1.37) | 0.337 | 1.02 (0.56 to 1.84) | 0.957 |
| BMI group ^b^ | Underweight or normal weight | N/A | N/A | N/A | N/A | 0.21 (0.01 to 4.32) | 0.308 | 4.95 (0.41 to 59.94) | 0.209 | N/A | N/A | N/A | N/A | N/A | N/A |
|  | Overweight | 0.39 (0.13 to 1.14) | 0.086 | 1.14 (0.40 to 3.27) | 0.808 | 2.16 (0.68 to 6.84) | 0.192 | 0.98 (0.32 to 2.98) | 0.976 | 3.23 (0.91 to 11.45) | 0.069 | **0.22 (0.06 to 0.86)** | **0.030** | 0.40 (0.05 to 3.54) | 0.411 |
|  | Obese | **1.44 (1.04 to 2.01)** | **0.029** | 1.04 (0.80 to 1.35) | 0.755 | 0.92 (0.71 to 1.19) | 0.517 | **1.57 (1.25 to 1.97)** | **<.001** | 1.24 (0.95 to 1.62) | 0.114 | 0.75 (0.54 to 1.06) | 0.106 | 0.86 (0.61 to 1.21) | 0.384 |
| Level of education | Middle school or lower education | **1.44 (1.02 to 2.03)** | **0.038** | 1.12 (0.84 to 1.49) | 0.438 | 0.88 (0.65 to 1.17) | 0.371 | **1.54 (1.17 to 2.01)** | **0.002** | 1.24 (0.88 to 1.75) | 0.226 | 0.70 (0.46 to 1.04) | 0.080 | 0.69 (0.43 to 1.11) | 0.129 |
|  | College or higher education | 1.39 (0.47 to 4.12) | 0.554 | 1.05 (0.62 to 1.79) | 0.850 | 1.19 (0.72 to 1.97) | 0.491 | **1.63 (1.10 to 2.42)** | **0.016** | 1.38 (0.91 to 2.12) | 0.134 | 0.76 (0.45 to 1.30) | 0.313 | 0.97 (0.58 to 1.62) | 0.899 |
| Household income | Lowest and second quartile | 1.30 (0.91 to 1.86) | 0.155 | 1.02 (0.77 to 1.35) | 0.910 | 0.91 (0.67 to 1.24) | 0.554 | **1.50 (1.14 to 1.97)** | **0.004** | 1.15 (0.84 to 1.59) | 0.388 | 0.82 (0.56 to 1.21) | 0.315 | 0.89 (0.60 to 1.31) | 0.539 |
|  | Third and highest quartile | 1.49 (0.82 to 2.71) | 0.194 | 1.22 (0.77 to 1.94) | 0.393 | 1.05 (0.68 to 1.63) | 0.818 | **1.65 (1.11 to 2.47)** | **0.015** | 1.53 (0.97 to 2.41) | 0.065 | 0.62 (0.36 to 1.07) | 0.089 | 0.80 (0.45 to 1.43) | 0.459 |
| Smoking status | Smoker or ex-smoker | 1.15 (0.63 to 2.10) | 0.655 | 1.28 (0.80 to 2.04) | 0.305 | 1.18 (0.76 to 1.84) | 0.462 | **1.51 (1.03 to 2.23)** | **0.035** | 1.19 (0.74 to 1.92) | 0.463 | 0.72 (0.40 to 1.29) | 0.264 | 0.79 (0.44 to 1.43) | 0.437 |
|  | Non-smoker | **1.43 (1.01 to 2.03)** | **0.045** | 1.00 (0.75 to 1.34) | 0.978 | 0.83 (0.62 to 1.12) | 0.226 | **1.57 (1.20 to 2.05)** | **0.001** | 1.31 (0.96 to 1.80) | 0.093 | 0.74 (0.50 to 1.09) | 0.124 | 0.86 (0.56 to 1.33) | 0.503 |

Abbreviations: BMI, body mass index; CI, confidence interval; KNHANES, Korea National Health and Nutrition Examination Survey; OR, odds ratio.

The values in bold font represent a significant variance (p<0.05).

^a^ According to guidelines from the National Institute for Health and Care Excellence, central adiposity is divided into three groups: healthy central adiposity (waist-to-height ratio 0.40–0.49), increased central adiposity (waist-to-height ratio 0.50–0.59), and high central adiposity (waist-to-height ratio ≥0.60).

^b^ According to the Asian-Pacific guidelines, BMI is divided into four groups: underweight (<18.5 kg/m^2^), normal (18.5–22.9 kg/m^2^),

overweight (23.0–24.9 kg/m^2^), and obese (25.0–34.9 kg/m^2^).

**Table S4**. Weighted odds ratios in the prevalence of type 2 diabetes mellitus, stratified by central adiposity groups, before and during the COVID–19 pandemic (weighted % [95% CI]).

| Variables | Central adiposity groups ^a^ | Overall (2005–2022) | | Pre-pandemic (2005–2019) | | During pandemic (2020–2022) | | Ratio of ORs (95% CI)  during the pandemic compared to before the pandemic (reference) | |
| --- | --- | --- | --- | --- | --- | --- | --- | --- | --- |
|  |  | Weighted OR (95% CI) | P-value | Weighted OR (95% CI) | P-value | Weighted OR (95% CI) | P-value | Weighted ratio of OR (95% CI) | P-value |
| **Sex** | |  |  |  |  |  |  |  |  |
| Male | Healthy central adiposity | 1.00 (ref) |  | 1.00 (ref) |  | 1.00 (ref) |  | 1.00 (ref) |  |
|  | Increased central adiposity | **3.40 (3.14 to 3.68)** | **<.001** | **3.24 (2.96 to 3.54)** | **<.001** | **3.60 (2.99 to 4.33)** | **<.001** | 1.11 (0.90 to 1.37) | 0.317 |
|  | High central adiposity | **7.55 (6.62 to 8.60)** | **<.001** | **6.87 (5.87 to 8.05)** | **<.001** | **7.58 (5.90 to 9.73)** | **<.001** | 1.10 (0.82 to 1.48) | 0.520 |
| Female | Healthy central adiposity | 1.00 (ref) |  | 1.00 (ref) |  | 1.00 (ref) |  | 1.00 (ref) |  |
|  | Increased central adiposity | **6.20 (5.63 to 6.84)** | **<.001** | **6.33 (5.67 to 7.07)** | **<.001** | **5.68 (4.60 to 7.02)** | **<.001** | 0.90 (0.71 to 1.14) | 0.376 |
|  | High central adiposity | **15.43 (13.80 to 17.25)** | **<.001** | **15.76 (13.88 to 17.89)** | **<.001** | **13.53 (10.64 to 17.19)** | **<.001** | 0.86 (0.65 to 1.13) | 0.269 |
| **Age, years** | |  |  |  |  |  |  |  |  |
| 30–39 | Healthy central adiposity | 1.00 (ref) |  | 1.00 (ref) |  | 1.00 (ref) |  | 1.00 (ref) |  |
|  | Increased central adiposity | **5.04 (3.75 to 6.78)** | **<.001** | **4.66 (3.42 to 6.35)** | **<.001** | **8.57 (2.82 to 26.04)** | **<.001** | 1.84 (0.58 to 5.82) | 0.301 |
|  | High central adiposity | **18.23 (12.46 to 26.67)** | **<.001** | **12.65 (8.29 to 19.30)** | **<.001** | **51.49 (15.99 to 165.78)** | **<.001** | **4.07 (1.17 to 14.12)** | **0.027** |
| 40–49 | Healthy central adiposity | 1.00 (ref) |  | 1.00 (ref) |  | 1.00 (ref) |  | 1.00 (ref) |  |
|  | Increased central adiposity | **2.89 (2.46 to 3.39)** | **<.001** | **2.58 (2.17 to 3.06)** | **<.001** | **5.23 (3.26 to 8.38)** | **<.001** | **2.03 (1.23 to 3.35)** | **0.006** |
|  | High central adiposity | **9.01 (7.11 to 11.41)** | **<.001** | **7.27 (5.50 to 9.61)** | **<.001** | **19.25 (11.55 to 32.11)** | **<.001** | **2.65 (1.48 to 4.74)** | **0.001** |
| 50–59 | Healthy central adiposity | 1.00 (ref) |  | 1.00 (ref) |  | 1.00 (ref) |  | 1.00 (ref) |  |
|  | Increased central adiposity | **2.17 (1.92 to 2.46)** | **<.001** | **2.13 (1.85 to 2.45)** | **<.001** | **2.30 (1.74 to 3.03)** | **<.001** | 1.08 (0.79 to 1.47) | 0.626 |
|  | High central adiposity | **4.44 (3.68 to 5.36)** | **<.001** | **3.96 (3.17 to 4.95)** | **<.001** | **5.52 (3.81 to 7.99)** | **<.001** | 1.39 (0.90 to 2.15) | 0.133 |
| ≥60 | Healthy central adiposity | 1.00 (ref) |  | 1.00 (ref) |  | 1.00 (ref) |  | 1.00 (ref) |  |
|  | Increased central adiposity | **1.75 (1.60 to 1.91)** | **<.001** | **1.67 (1.52 to 1.84)** | **<.001** | **1.98 (1.63 to 2.39)** | **<.001** | 1.18 (0.96 to 1.47) | 0.125 |
|  | High central adiposity | **2.66 (2.40 to 2.96)** | **<.001** | **2.61 (2.32 to 2.94)** | **<.001** | **2.75 (2.18 to 3.47)** | **<.001** | 1.05 (0.81 to 1.37) | 0.697 |
| **Region of residence** | |  |  |  |  |  |  |  |  |
| Urban | Healthy central adiposity | 1.00 (ref) |  | 1.00 (ref) |  | 1.00 (ref) |  | 1.00 (ref) |  |
|  | Increased central adiposity | **4.58 (4.27 to 4.91)** | **<.001** | **4.40 (4.07 to 4.75)** | **<.001** | **4.90 (4.17 to 5.77)** | **<.001** | 1.12 (0.93 to 1.34) | 0.232 |
|  | High central adiposity | **10.99 (10.04 to 12.03)** | **<.001** | **10.36 (9.33 to 11.50)** | **<.001** | **11.46 (9.50 to 13.84)** | **<.001** | 1.11 (0.89 to 1.37) | 0.354 |
| Rural | Healthy central adiposity | 1.00 (ref) |  | 1.00 (ref) |  | 1.00 (ref) |  | 1.00 (ref) |  |
|  | Increased central adiposity | **3.41 (3.00 to 3.87)** | **<.001** | **3.42 (2.96 to 3.96)** | **<.001** | **3.02 (2.27 to 4.03)** | **<.001** | 0.88 (0.64 to 1.22) | 0.451 |
|  | High central adiposity | **6.65 (5.68 to 7.78)** | **<.001** | **6.84 (5.71 to 8.20)** | **<.001** | **5.06 (3.72 to 6.89)** | **<.001** | 0.74 (0.52 to 1.06) | 0.097 |
| **BMI group ^b^** | |  |  |  |  |  |  |  |  |
| Underweight or normal weight | Healthy central adiposity | 1.00 (ref) |  | 1.00 (ref) |  | 1.00 (ref) |  | 1.00 (ref) |  |
|  | Increased central adiposity | **4.04 (3.67 to 4.44)** | **<.001** | **3.69 (3.31 to 4.12)** | **<.001** | **5.34 (4.37 to 6.53)** | **<.001** | **1.45 (1.15 to 1.82)** | **0.002** |
|  | High central adiposity | 1.25 (0.58 to 2.72) | 0.573 | 0.64 (0.27 to 1.52) | 0.313 | **15.44 (3.30 to 72.30)** | **<.001** | **24.13 (4.10 to 141.87)** | **<.001** |
| Overweight | Healthy central adiposity | 1.00 (ref) |  | 1.00 (ref) |  | 1.00 (ref) |  | 1.00 (ref) |  |
|  | Increased central adiposity | **4.88 (4.27 to 5.57)** | **<.001** | **4.33 (3.75 to 5.00)** | **<.001** | **8.03 (5.63 to 11.47)** | **<.001** | **1.86 (1.27 to 2.72)** | **0.002** |
|  | High central adiposity | **7.51 (5.48 to 10.28)** | **<.001** | **6.16 (4.25 to 8.94)** | **<.001** | **15.88 (8.24 to 30.60)** | **<.001** | **2.58 (1.21 to 5.48)** | **0.014** |
| Obese | Healthy central adiposity | 1.00 (ref) |  | 1.00 (ref) |  | 1.00 (ref) |  | 1.00 (ref) |  |
|  | Increased central adiposity | **4.94 (3.78 to 6.46)** | **<.001** | **4.66 (3.49 to 6.23)** | **<.001** | **5.81 (2.82 to 11.97)** | **<.001** | 1.25 (0.57 to 2.72) | 0.580 |
|  | High central adiposity | **12.30 (9.38 to 16.13)** | **<.001** | **11.33 (8.44 to 15.20)** | **<.001** | **14.57 (7.05 to 30.14)** | **<.001** | 1.29 (0.59 to 2.82) | 0.528 |
| **Level of education** | |  |  |  |  |  |  |  |  |
| College or higher education | Healthy central adiposity | 1.00 (ref) |  | 1.00 (ref) |  | 1.00 (ref) |  | 1.00 (ref) |  |
|  | Increased central adiposity | **3.26 (2.96 to 3.59)** | **<.001** | **3.16 (2.85 to 3.51)** | **<.001** | **3.40 (2.60 to 4.45)** | **<.001** | 1.08 (0.81 to 1.43) | 0.623 |
|  | High central adiposity | **5.74 (5.12 to 6.44)** | **<.001** | **5.85 (5.15 to 6.63)** | **<.001** | **4.40 (3.30 to 5.87)** | **<.001** | 0.75 (0.55 to 1.03) | 0.076 |
| High school or lower education | Healthy central adiposity | 1.00 (ref) |  | 1.00 (ref) |  | 1.00 (ref) |  | 1.00 (ref) |  |
|  | Increased central adiposity | **4.10 (3.77 to 4.45)** | **<.001** | **3.96 (3.60 to 4.36)** | **<.001** | **4.13 (3.49 to 4.90)** | **<.001** | 1.04 (0.86 to 1.27) | 0.679 |
|  | High central adiposity | **9.85 (8.69 to 11.16)** | **<.001** | **8.97 (7.69 to 10.46)** | **<.001** | **10.07 (8.05 to 12.61)** | **<.001** | 1.12 (0.86 to 1.47) | 0.403 |
| **Household income** | |  |  |  |  |  |  |  |  |
| Third and highest quartile | Healthy central adiposity | 1.00 (ref) |  | 1.00 (ref) |  | 1.00 (ref) |  | 1.00 (ref) |  |
|  | Increased central adiposity | **4.17 (3.83 to 4.55)** | **<.001** | **3.88 (3.53 to 4.27)** | **<.001** | **5.06 (4.12 to 6.21)** | **<.001** | **1.30 (1.04 to 1.64)** | **0.022** |
|  | High central adiposity | **8.60 (7.74 to 9.56)** | **<.001** | **8.24 (7.32 to 9.29)** | **<.001** | **8.88 (7.02 to 11.23)** | **<.001** | 1.08 (0.83 to 1.40) | 0.582 |
| Lowest and Second quartile | Healthy central adiposity | 1.00 (ref) |  | 1.00 (ref) |  | 1.00 (ref) |  | 1.00 (ref) |  |
|  | Increased central adiposity | **4.23 (3.88 to 4.62)** | **<.001** | **4.27 (3.87 to 4.71)** | **<.001** | **3.86 (3.20 to 4.65)** | **<.001** | 0.90 (0.73 to 1.12) | 0.350 |
|  | High central adiposity | **9.98 (8.76 to 11.37)** | **<.001** | **9.18 (7.86 to 10.73)** | **<.001** | **9.90 (7.75 to 12.65)** | **<.001** | 1.08 (0.81 to 1.44) | 0.608 |
| **Smoking status** | |  |  |  |  |  |  |  |  |
| Non-smoker | Healthy central adiposity | 1.00 (ref) |  | 1.00 (ref) |  | 1.00 (ref) |  | 1.00 (ref) |  |
|  | Increased central adiposity | **2.90 (2.67 to 3.16)** | **<.001** | **2.74 (2.49 to 3.01)** | **<.001** | **3.32 (2.74 to 4.03)** | **<.001** | 1.21 (0.98 to 1.50) | 0.078 |
|  | High central adiposity | **6.64 (5.84 to 7.57)** | **<.001** | **6.18 (5.31 to 7.19)** | **<.001** | **7.07 (5.46 to 9.15)** | **<.001** | 1.14 (0.85 to 1.54) | 0.378 |
| Current and ex- smoker | Healthy central adiposity | 1.00 (ref) |  | 1.00 (ref) |  | 1.00 (ref) |  | 1.00 (ref) |  |
|  | Increased central adiposity | **6.00 (5.46 to 6.59)** | **<.001** | **6.11 (5.49 to 6.78)** | **<.001** | **5.42 (4.43 to 6.64)** | **<.001** | 0.89 (0.71 to 1.12) | 0.307 |
|  | High central adiposity | **14.91 (13.38 to 16.63)** | **<.001** | **15.24 (13.45 to 17.26)** | **<.001** | **12.76 (10.19 to 15.97)** | **<.001** | 0.84 (0.65 to 1.08) | 0.175 |

Abbreviations: BMI, body mass index; CI, confidence interval; KNHANES, Korea National Health and Nutrition Examination Survey; OR, odds ratio.

The values in bold font represent a significant variance (p<0.05).

^a^ According to guidelines from the National Institute for Health and Care Excellence, central adiposity is divided into three groups: healthy central adiposity (waist-to-height ratio 0.40–0.49), increased central adiposity (waist-to-height ratio 0.50–0.59), and high central adiposity (waist-to-height ratio ≥0.60).

^b^ According to the Asian-Pacific guidelines, BMI is divided into four groups: underweight (<18.5 kg/m^2^), normal (18.5–22.9 kg/m^2^),

overweight (23.0–24.9 kg/m^2^), and obese (25.0–34.9 kg/m^2^).
